# State-dependent connectivity in auditory-reward networks predicts peak pleasure experiences to music

**DOI:** 10.1371/journal.pbio.3002732

**Published:** 2024-08-12

**Authors:** Kazuma Mori, Robert Zatorre

**Affiliations:** 1 Institute for Quantum Life Science, National Institutes for Quantum Science and Technology, Chiba, Japan; 2 Center for Information and Neural Networks (CiNet), National Institute of Information and Communications Technology (NICT), Osaka, Japan; 3 Montréal Neurological Institute, McGill University, Montréal, Canada; 4 International Laboratory for Brain, Music and Sound Research, Montréal, Canada; 5 Centre for Research in Brain, Language and Music, Montréal, Canada; New York University, UNITED STATES OF AMERICA

## Abstract

Music can evoke pleasurable and rewarding experiences. Past studies that examined task-related brain activity revealed individual differences in musical reward sensitivity traits and linked them to interactions between the auditory and reward systems. However, state-dependent fluctuations in spontaneous neural activity in relation to music-driven rewarding experiences have not been studied. Here, we used functional MRI to examine whether the coupling of auditory-reward networks during a silent period immediately before music listening can predict the degree of musical rewarding experience of human participants (*N* = 49). We used machine learning models and showed that the functional connectivity between auditory and reward networks, but not others, could robustly predict subjective, physiological, and neurobiological aspects of the strong musical reward of chills. Specifically, the right auditory cortex-striatum/orbitofrontal connections predicted the reported duration of chills and the activation level of nucleus accumbens and insula, whereas the auditory-amygdala connection was associated with psychophysiological arousal. Furthermore, the predictive model derived from the first sample of individuals was generalized in an independent dataset using different music samples. The generalization was successful only for state-like, pre-listening functional connectivity but not for stable, intrinsic functional connectivity. The current study reveals the critical role of sensory-reward connectivity in pre-task brain state in modulating subsequent rewarding experience.

## Abstract 2

Listening to music does not always elicit the same emotional response in the same person because that person’s cognitive state can change, impacting their response. This study reveals that pleasure from music is in part determined by the state of resting auditory-reward brain networks prior to listening.

## Introduction

Music listening is an important part of everyday life for the vast majority of society [1]. One of the main reasons for music listening is to experience pleasure and reward [2,3]. Previous studies have consistently reported that when people experience pleasure in music, the auditory cortices and the mesolimbic reward circuitry are activated [4–8]. Furthermore, the functional interaction between these systems increases as a function of the hedonic value of music [9]. Stable individual differences in music reward sensitivity traits are also linked to interactions between auditory and reward brain networks [10–12]. However, music may not always elicit the same emotional response in the same person because that person’s cognitive state can change, which impacts their emotional response [13]. The change in cognitive state may reflect the pre-listening brain activities. For example, when people look forward to waiting to listen to their favorite music, the momentary brain networks during the period may differ from daily stable brain networks. Although such state-dependent brain fluctuations have not been studied in the field of musical reward studies, it is possible that the pre-listening brain network is tied to the musical reward experience.

The resting brain network before music listening could be a determinant of musical pleasure, based on other literature. In the past decade, many studies have reported that 5 to 10 min of intrinsic neural network activity, i.e., resting-state functional connectivity (RSFC), could predict various human abilities or traits [14–17]. However, questions have also been raised about the sources of this predictive ability [18,19]. On the other hand, recent studies indicated that the short (several seconds to dozen seconds) resting brain network before an experimental task could predict cognitive task performance [20–22], mind wandering [23], and value-based decision-making [24]. These studies suggest that the state-endogenous fluctuation of the brain immediately before the task influences subsequent neural activity, and, hence, behavior. Because the functional link between the auditory and reward brain regions during music listening positively correlates with the reward strength [7,9,10,25], emotional response to music may be facilitated when the auditory-reward brain network is in a relatively higher state of interaction prior to the musical experience.

Anticipation of an upcoming piece at rest would modulate the connection between auditory and reward brain regions. Past studies reported that the anticipation of future rewards activates rewarding brain regions, especially the ventromedial prefrontal cortex (vmPFC) [26–30], as well as nucleus accumbens (NAcc), and amygdala [31,32]. These brain activities should reflect that people enjoy the moments leading up to the reward [33]. Moreover, anticipations for the next musical item may have an effect on auditory brain activity at rest, probably because of imagination and memory about music [34–36]. Therefore, the synchronization of auditory and rewarding brain regions could change by anticipatory brain activity during the resting period, resulting in an enhanced emotional response to music.

**Fig 1:**
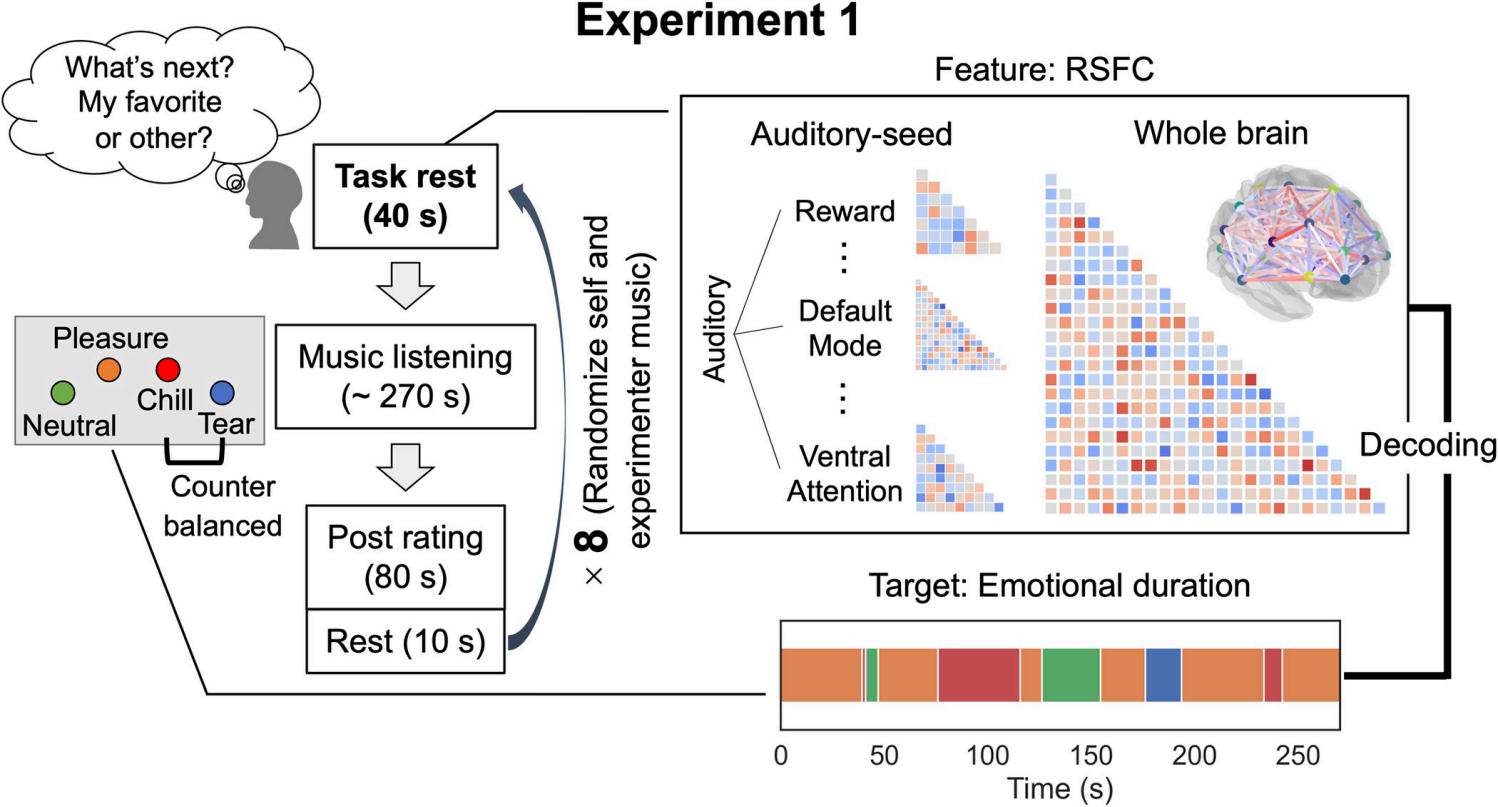
Experimental 1 task and examined variables. The assumption of participants’ anticipation of music during the task rest is validated, as shown in S1 Fig. RSFC was calculated from the task rest period each for 8 pieces. We used both auditory-seed and whole-brain RSFC features (see Materials and methods). In the listening experiment, participants always reported one of the 4 emotions. Using the RSFC features and each of the 4 emotional durations, we performed machine learning (i.e., brain decoding) analysis. After the listening, participants rated their emotional state. We set 10-s rest before task rest to avoid the remaining rating effect. RSFC, resting state functional connectivity.

In the present study, we aim to reveal whether the resting brain state immediately before music listening is specifically associated with a rewarding experience. To examine this question, we recruited 49 participants for functional MRI (fMRI) experiments and simultaneous psychophysiological measurements (Experiment 1: 38, Experiment 2: 11). Participants listened to their favorite and experimenter-selected music. Listeners reported their emotional state behaviorally in real time by indicating if they were experiencing neutral, pleasure, chills (defined by goosebumps, shivers), or tears (defined by weeping, lump in the throat) responses [37,38]. Although chills are known to be an intense pleasure [5,25,39], tears are mixed sad and pleasure experience [37,38,40]. Therefore, we speculated that the different pre-listening brain networks are reflected in responses to pleasure/chills and tears. Participants were instructed that they would listen to their favorite music or other music in the experiment. Therefore, they knew that they would listen to highly rewarding music, but they did not know which pieces would play (Figs 1 and S1). We used not only chills- and tears-evoked music but also experimenter-selected songs because we thought that increasing uncertainty of the musical stimuli presented in the experiment may enhance anticipation for the next music. In line with a recent technical advance in neuroimaging research to identify FC patterns associated with complex cognitive functions [22,23,41], we evaluated RSFC predictability by machine learning models.

## Results

### Prediction of emotional responses to music from RSFC immediately before music listening

**Fig 2:**
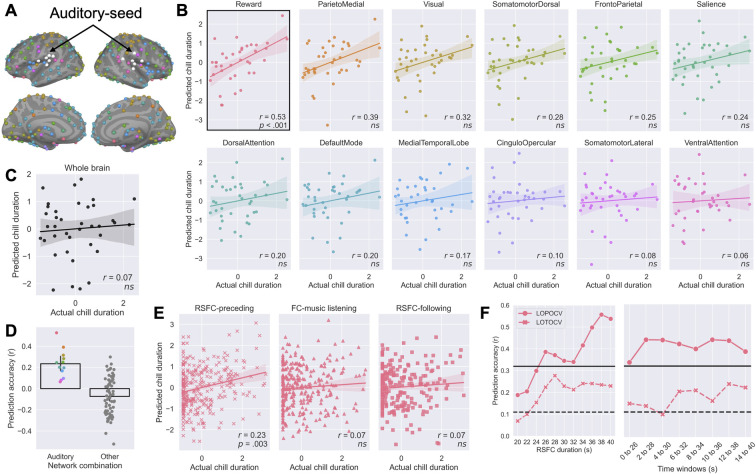
Task RSFC predictive modeling of subjective chills duration. (**A**) A total of 288 ROI points including cortical and subcortical regions in 13 networks from [42,45]. Different colors represent the different networks. White circles correspond to auditory areas [45] used as seeds for analysis. (**B**) Performance of predictive model by auditory-seed RSFC immediately before music listening. Each panel shows the results of the LOPOCV prediction using the different combinations of auditory and other networks. The actual versus predicted chill durations are shown in the plots. Each dot represents 1 participant. Only the auditory-reward network correlation remained significant after FDR correction. (**C**) Performance of whole brain predictive model. (**D**) Model performance comparison between the 12 auditory-related network combinations and the other 66 network combinations. Each dot represents 1 prediction score. (**E**) Predictive performance of the auditory-reward model by the LOTOCV. The left panel shows the prediction result of chills duration from auditory-reward RSFC on a per-trial basis. The center panel shows the prediction result of chills duration by FC during music listening. The right panel shows the prediction result of chills duration to the previous music by subsequent RSFC. Each dot represents each trial. (**F**) (Left) Auditory-reward predictive model performance as a function of the different time duration windows of RSFC immediately before music listening. (Right) The prediction accuracy of the auditory-reward RSFC over successive 26-s windows from resting start point to 40 s. Solid and dashed lines show uncorrected *p* = .05 for LOPOCV and LOTOCV, respectively. FC, functional connectivity; FDR, false discovery rate; LOPOCV, leave-one-participant-out cross-validation; LOTOCV, leave-one-trial-out cross-validation; ROI, region of interest; RSFC, resting state functional connectivity. Error bands and bars indicating the 95% confidence interval. Underlying data and scripts are available at https://doi.org/10.17605/OSF.IO/TNRXE and in S1 Data.

We applied machine learning analysis to identify whether the 40-s RSFC between auditory cortex seeds (defined independently from [42]) and 12 separate resting networks (Fig 2A) could predict the duration of self-reported emotional responses during music listening. We performed a linear machine learning analysis for each of the 4 emotional durations (not an emotion classifier). The duration of emotional responses across trials is strongly tied to the global emotion intensity ratings after music listening (Pleasure: *r*_*302*_ = 0.43, *p* < .001, Chill: *r*_*302*_ = 0.66, *p* < .001, Tear: *r*_*302*_ = 0.70, *p* < .001). We selected the duration of online subjective experience rather than intensity rating after music as a dependent variable because we also examined physiological and neural activity during the online experience (see below). To analyze the target variables in a uniform format, the duration of subjective experience is essential for the current study.

To evaluate the prediction performance, we applied the least absolute shrinkage and selection operator (LASSO) machine learning models [43] to data from test participants using leave-one-participant-out cross-validation (LOPOCV) (see Materials and methods). Only the auditory-reward network RSFC model showed statistically significant prediction for subjective durations of chills during music listening following the resting-state period. Specifically, the correlation between predicted and actual chills duration was Pearson’s *r*_*36*_ = 0.53 (false discovery rate (FDR) correction, permutation test, *p* < .001), whereas other auditory-seed networks did not show significant correlations (Fig 2B). No RSFC auditory-seed network could significantly predict durations of reported neutral, pleasure, or tear responses (S3 Fig). Further, the machine learning model by auditory-reward network RSFC did not show a significant prediction for the number of chills (*r*_*36*_ = .14), emphasizing the RSFC predictive ability for the duration of musical chills. The result also suggests that the results are not related to the amount of motor activity associated with the number of button presses. Note that the pleasure ratings obtained after the end of the excerpt (range 1 to 4) were high both for self-selected (*M* = 3.17, *SD* = 0.74, specifically, chill music: *M* = 3.37, *SD* = 0.75, tear music: *M* = 2.99, *SD* = 0.68) and experimenter-selected (*M* = 3.01, *SD* = 0.77) music. Participants felt high pleasure during music listening; therefore, the musical chills reflected a pleasurable experience (as opposed to some other arousal-related emotion).

Whole-brain RSFC could not predict durations of reported chill (Fig 2C) nor neutral, pleasure, or tear responses (S3 Fig). Furthermore, as an exploratory approach, we examined all 78 possible network combinations derived from 13 networks to predict the subjective duration of chills (Fig 2D). We found that the auditory-reward network had the best predictive ability compared to the 77 other pairs of networks. In addition, BOLD signal magnitude patterns in the pre-listening silent period did not predict the duration of chills (S1 Text and Figs 1 and 6). The auditory-reward network without removing the physiological signals also did not predict the duration of chills (S5 Fig), although we removed the physiological signal effect for RSFC in the main analysis due to the strong noise source [44] (see Materials and methods).

### Trial-by-trial chills experience prediction by auditory-reward RSFC with different time periods

As a complement to the previous per-participant analysis, we carried out a trial-by-trial machine learning analysis of whether the auditory-reward RSFC predicts the duration of chills. To reveal the specificity of the FC predictive ability for the subjective chills experience, we made 3 machine learning models setting RSFC preceding music, FC during music listening, and RSFC following music as features. We used the overall music-listening period for the FC during music listening, not just during the chill periods. Because chill periods were sometimes very short and inconsistent for every participant, it was challenging to estimate FC steadily. Therefore, we used the overall music-listening period to compare the predictive ability of the other 2 periods fairly.

Using leave-one-trial-out cross-validation (LOTOCV), we found that the auditory-reward RSFC preceding music significantly predicted the duration of the chill on a per-trial basis, *r*_*296*_ = 0.23 (FDR correction, permutation test, *p* < .001) (Fig 2E-left). But neither the FC during music listening nor the RSFC following music listening could predict the duration of the chill (Fig 2E-middle, right). These results indicated that the auditory-reward brain network in the resting state prior to music is important in predicting subsequent emotional responses. In addition, the correlation between predicted chills duration by the auditory-reward RSFC preceding music and music type (coding self: 1 and experimenter: 2) did not reach significance (*r*_*296*_ = −0.06). Thus, the predictive score did not reflect self- and experimenter-music order (see more detail in S4 Fig).

### Validation of subjective chills prediction by auditory-reward RSFC

To further determine the sensitivity of the auditory-reward RSFC to predict the duration of subjective chills, we examined the predictive performance using different RSFC cumulative durations before music listening. When the duration of RSFC was more than 26 s, the auditory-reward RSFC significantly predicted the duration of chills for LOPOCV and LOTOCV analysis (uncorrected *p*s < .05, Fig 2F-left). The prediction accuracy increased linearly from 20 to 40 s (LOPOCV: *r*^*2*^_*9*_ = 0.88, *p* < .001, LOTOCV: *r*^*2*^_*9*_ = 0.53, *p* = .01). The successful prediction by RSFC duration of 26 s corresponds well with a previous report [22]. Using the minimum significant duration of the auditory-reward RSFC, we tried to predict the duration of subjective chills by successive 26-s sliding windows from 0 to 40 s, to investigate the time frame difference for prediction. The prediction accuracy was always significant (uncorrected *p*s < .05, Fig 2F-right) except from 4 to 30 s for LOTOCV (*p* = .077). These findings validate the robust predictive ability for the duration of the chills by the auditory-reward RSFC immediately before music listening.

### Prediction of physiological and neural activity during chills experience by auditory-reward RSFC

**Fig 3:**
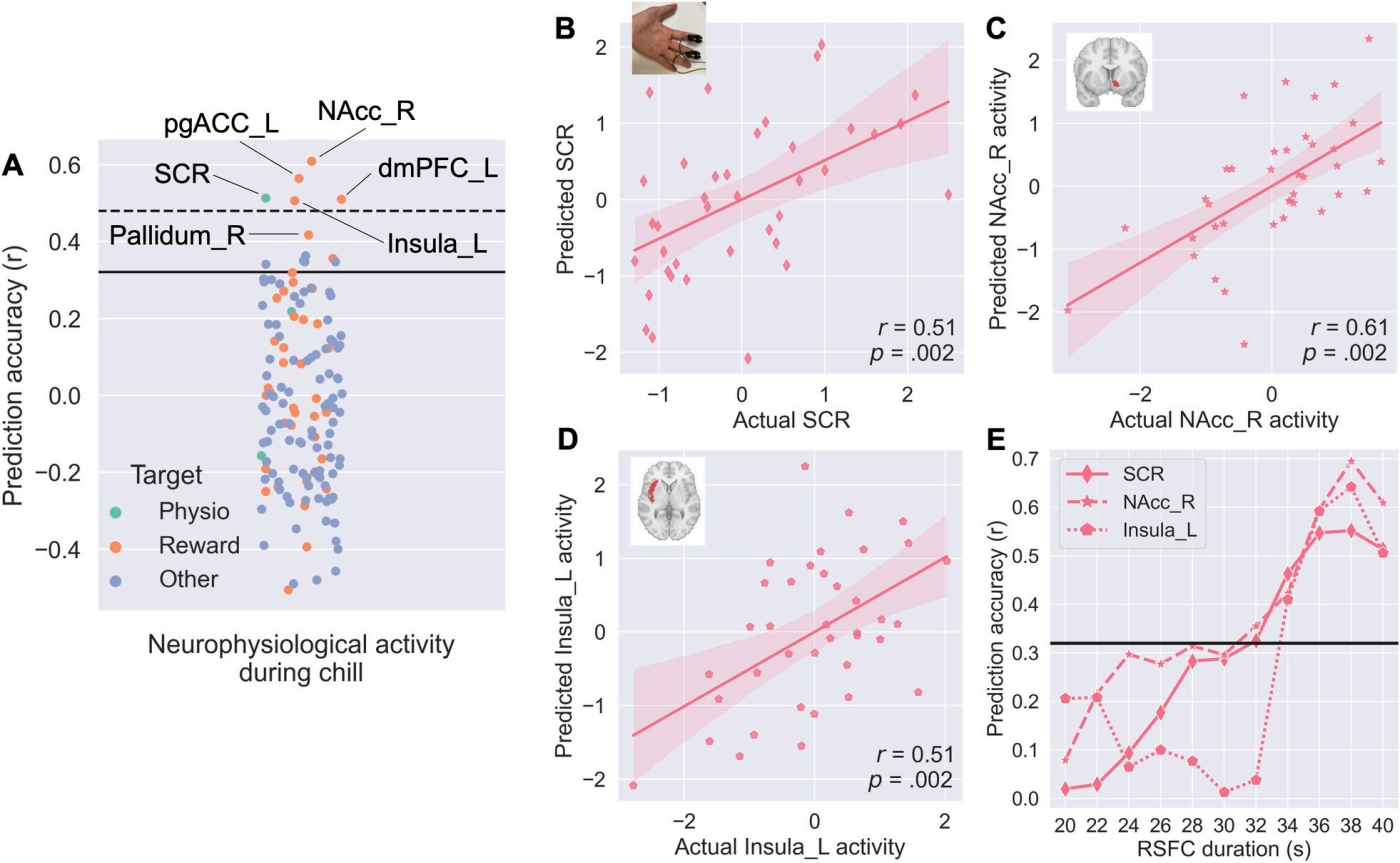
Task auditory-reward RSFC predictive modeling for physiological and neural responses during musical chills using LOPOCV. (**A**) Prediction accuracy for physiological (SCR, HR, RR) and neural (140 ROIs from AAL3.1 [47]) activities during chills experience. Reward-related regions were defined by [49]. Solid and dashed lines show *p* = .05 for uncorrected and FDR corrected, respectively. (**B**) The actual versus predicted SCR intensity during chills is shown in the plots. Each dot represents 1 participant. (**C**) The actual versus predicted right NAcc activity during chills is shown in the plot. (**D**) The actual versus predicted left insula activity during chills is shown in the plots. (**E**) The auditory-reward predictive model performance as a function of different time duration windows of RSFC for the above 3 indexes. dmPFC, dorsomedial prefrontal cortex (Frontal-Sup-Medial from AAL3.1); FDR, false discovery rate; HR, heart rate; L, left; LOPOCV, leave-one-participant-out cross-validation; NAcc, nucleus accumbens; pgACC, pregenual anterior cingulate cortex; R, right; ROI, region of interest; RR, respiration rate; RSFC, resting state functional connectivity; SCR, skin conductance response. Error bands indicating the 95% confidence interval. Underlying data and scripts are available at https://doi.org/10.17605/OSF.IO/TNRXE and in S1 Data.

Subjective chills experiences are typically accompanied by physiological arousal and brain activity in reward regions [4,5,37,38]. Skin conductance response (SCR) is a robust physiological marker of the arousal effect of chills [46], and several studies suggested that the mesolimbic pathway, especially right NAcc activity, is critical for brain activity of chills [5,9,10]. We investigated whether the auditory-reward RSFC immediately before music listening can predict these neurophysiological aspects of chills by making the LASSO-LOPOCV predictive model. We tried to predict the sum of the cumulative intensity of SCR, heart rate (HR), respiration rate (RR), and the BOLD signal in specific brain regions during the musical chills experience. The BOLD signal was extracted from each of the 140 region of interests (ROIs) [47]. As shown in Fig 3A, several neurophysiological activities during the chills experience can be predicted well. Specifically, the auditory-reward RSFC significantly predicts SCR (*r*_*36*_ = 0.51, *p* = .002) (Fig 3B), but not HR or RR. The same network also predicted BOLD activity in the right NAcc (*r*_*36*_ = 0.61, *p* = .002), left insula (*r*_*36*_ = 0.51, *p* = .002) (Fig 3C and 3D), left pgACC (*r*_*36*_ = 0.56, *p* = .002), and dmPFC (*r*_*36*_ = 0.51, *p* = .004) (S7 Fig) (FDR correction, permutation test). The right NAcc activity during chills positively correlated with the left insula activity during chills (*r*_*36*_ = 0.56, *p* < .001); therefore, the auditory-reward RSFC predicts co-activation of NAcc and insula. These results indicate that not only subjective chills experience but also physiological and neural aspects associated with pleasurable chills could be predicted by the auditory-reward RSFC.

Similar to subjective chills prediction, we examined the predictive performance of different RSFC durations to validate the predictive ability of the auditory-reward RSFC model on physiological and neural measures. The result showed that when the duration of RSFC was more than 34 s, the auditory-reward RSFC significantly predicted all the SCR, right NAcc, and left insula activity (uncorrected *p*s < .05, Fig 3E-left), indicating the robust predictive ability of the auditory-reward RSFC again. The prediction accuracy linearly increased from 20 to 40 s for SCR (*r*^*2*^_*9*_ = 0.95, *p* < .001) and right NAcc (*r*^*2*^_*9*_ = 0.88, *p* < .001), but the linear effect is weaker for the left insula (*r*^*2*^_*9*_ = 0.48, *p* = .018).

Finally, we investigated which auditory regions are most important for prediction of physiological and neural response by distinguishing between primary and secondary auditory regions from HCP ROIs [48]. We performed a similar machine learning analysis as above. We found that the RSFC between the bilateral primary auditory region and reward can predict SCR activity, whereas the right secondary auditory-reward RSFC can predict the right NAcc activity (S8 Fig).

### Asymmetry of the auditory-reward RSFC and behavioral relevance

**Fig 4:**
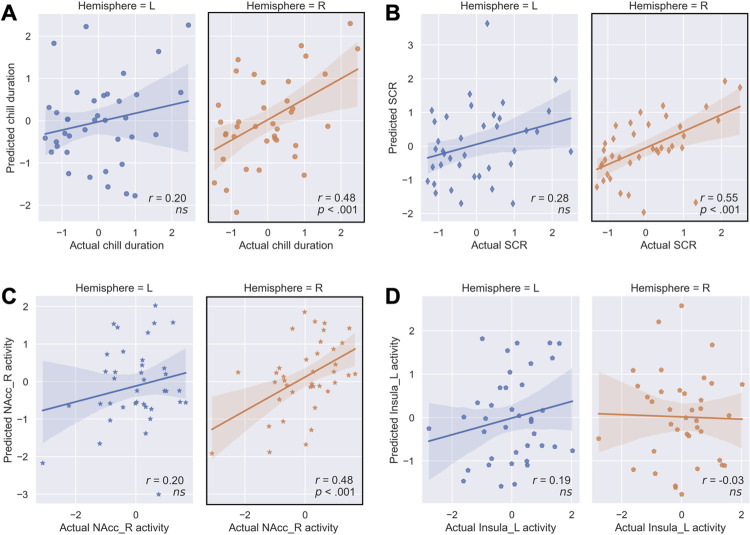
Performance of left or right auditory-reward RSFC predictive modeling for the (**A**) subjective duration, (**B**) SCR intensity, (**C**) right NAcc activity, and (**D**) left insula activity of chills by LOPOCV; L, left; LOPOCV, leave-one-participant-out cross-validation; NAcc, nucleus accumbens; R, right; RSFC, resting-state functional connectivity; SCR, skin conductance response. Error bands indicating the 95% confidence interval. Underlying data and scripts are available at https://doi.org/10.17605/OSF.IO/TNRXE and in S1 Data.

Many previous studies indicated that emotional responses to music are related to the functional interactions between reward regions in the right more than the left hemisphere [7,9,10,25]. To test for the asymmetric effect in the auditory-reward RSFC, we performed a machine learning analysis on whether the RSFC within each hemisphere has meaningful relationships to the chills experience. We confirmed that the auditory-reward RSFC from the right hemisphere, but not the left hemisphere, could predict the subjective duration of chills (*r*_*36*_ = 0.48, *p* < .001) (Fig 4A), the intensity of SCR (*r*_*36*_ = 0.55, *p* < .001) (Fig 4B) and right NAcc activity (*r*_*36*_ = 0.48, *p* < .001) (Fig 4C) with permutation test and FDR correction. Neither hemisphere significantly predicts left insula activity. To examine the statistical hemisphere difference of predictability, we performed a bootstrap test (see Materials and methods). Although the prediction accuracy of the model on subjective chills and SCR failed to reach significance (bootstrap 95% CI = [−0.11, 0.66] and [−0.11, 0.59], respectively, *p*s > .05), the prediction accuracy on the right NAcc BOLD signal showed a significant difference (bootstrap 95% CI = [0.00, 0.60], *p* = .05), indicating that the right hemisphere has a high predictive ability.

### The predictive weight of auditory-reward RSFC for the subjective, physiological, and neural responses of chills

**Fig 5:**
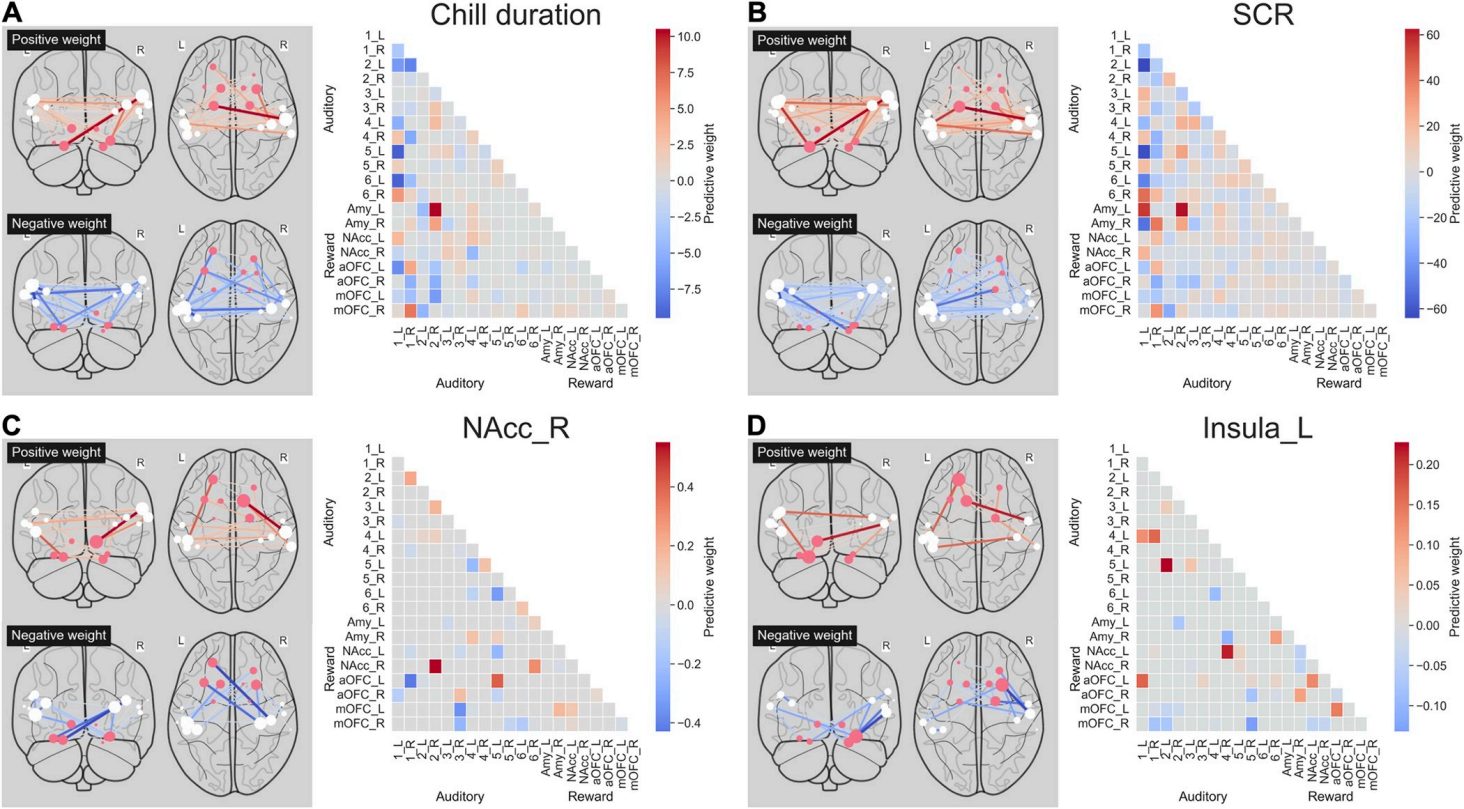
The predictive weight of auditory-reward RSFC for subjective, physiological, and neural responses to chills from LOPOCV. (Left) Coronal and axial views of connections in the transparent brain. White and magenta circles correspond to auditory and reward areas, respectively. Nodes are sized according to the sum of edges in the positive and negative weights (i.e., larger nodes had more edges overall) and colored according to the difference between edges in the positive and negative networks (i.e., red nodes had mostly positive edges and blue nodes had mostly negative edges). (Right) The matrices showing the prediction weights for every pair of ROIs are also provided. Predictive weight for (**A**) subjective chills duration, (**B**) SCR intensity, (**C**) right NAcc activity, and (**D**) left insula activity. a, anterior; Amy, amygdala; L, left; LOPOCV, leave-one-participant-out cross-validation; m, medial; R, right; NAcc, nucleus accumbens; OFC, orbitofrontal cortex; ROI, region of interest; RSFC, resting-state functional connectivity; SCR, skin conductance response. Underlying data and scripts are available at https://doi.org/10.17605/OSF.IO/TNRXE and in S1 Data.

With the applied machine learning pipeline, nonzero LASSO regression weights delineate the predictive network. Each weight can be interpreted as the relative importance of the connectivity in the prediction. Positive (negative) weights translate to stronger interregional functional connectivity predicting longer (shorter) duration of chills experience. The predictive network for the subjective chill duration (Fig 5A), SCR intensity (Fig 5B), right NAcc intensity (Fig 5C), and left insula intensity (Fig 5D) of chills are depicted in the plot.

For the subjective chill duration, the strongest positive predictive connections were found between one of the right auditory regions and the left amygdala. However, as shown in Fig 5A, the cumulative positive weight (circle size) was also high in the amygdala, NAcc, and mOFC. Both positive and negative cumulative weights were high in the auditory region. These results indicated that the overall auditory-reward network is important for predicting subjective chills experience.

The predictive weight for physiological and neural activity during chills showed the importance of specific reward regions. The higher positive predictive weight for SCR (Fig 5B) was found between the bilateral auditory region and the left amygdala. The cumulative positive weight indicated the importance of the left amygdala, whereas both positive and negative cumulative weight was high in the auditory region. For the right NAcc and the left insula activities (Fig 5C and 5D), the right auditory regions showed a higher positive predictive weight with the right and left NAcc, respectively. Moreover, for the right NAcc and the left insula, the left auditory regions showed a higher positive predictive weight with the left aOFC. In contrast, the negative predictive weight for right NAcc was mainly found between the auditory regions and a/mOFC, and weight for left insula was found between the auditory regions and right mOFC. These findings indicated that the RSFC between the auditory areas and amygdala is critical for predicting the physiological arousal of chills, whereas the RSFC between the auditory and NAcc/OFC is important to predicting rewarding brain activity of chills (see the similar connectivity weight for the right hemisphere prediction in S9 Fig).

### Generalization of the predictive model for independent experimental data

**Fig 6:**
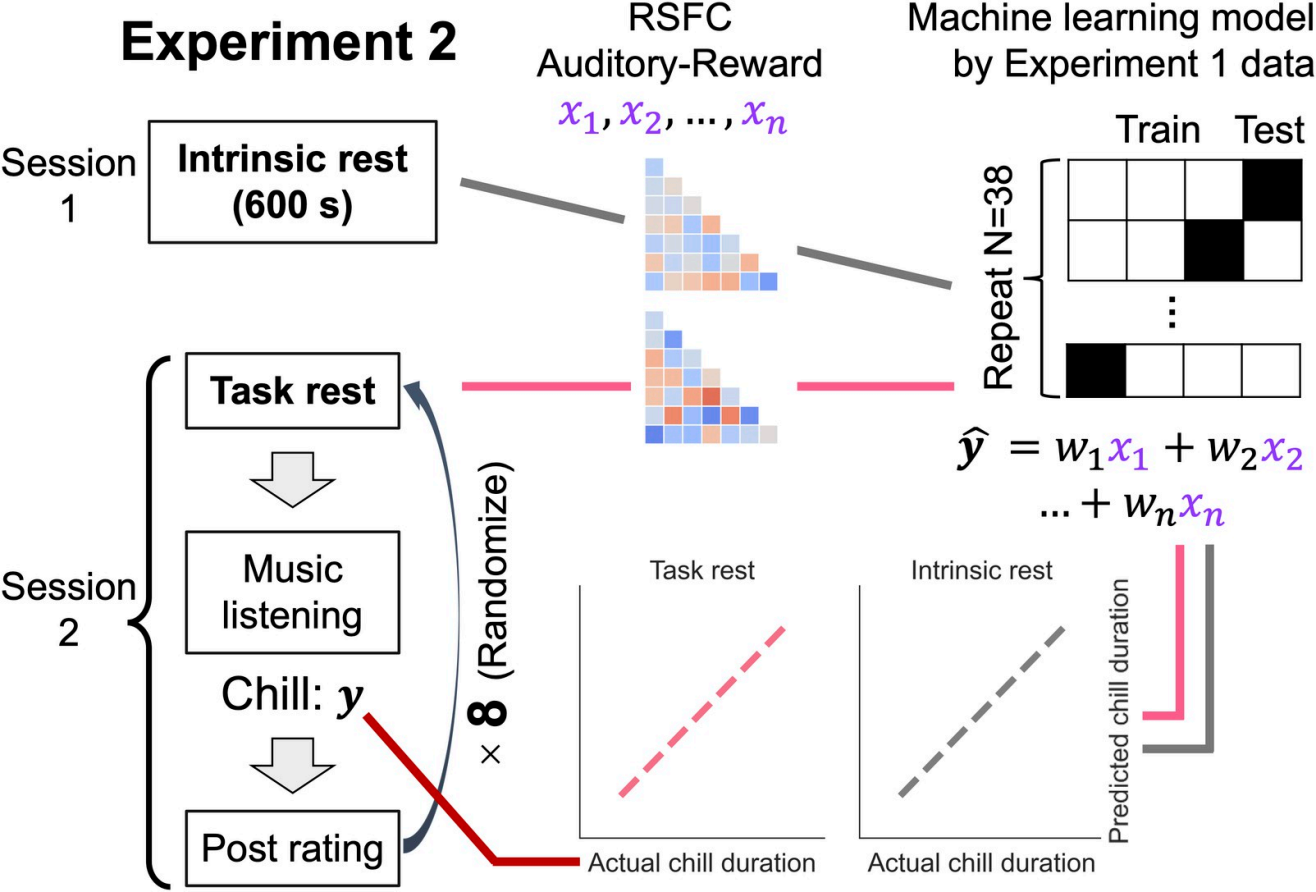
Experiment 2 tasks and predictive model generalization procedures. We conducted 2 sessions: one recorded intrinsic rest with no tasks, and the other was similar to Experiment 1. We calculated the auditory-reward RSFC for intrinsic and task rest, respectively. The predicted chills duration (y-hat score) was obtained by dot-products of the auditory-reward RSFC in Experiment 2 and the weights of the machine learning model made in Experiment 1 dataset. To evaluate the generalized predictive ability, we examined the correlation between actual chills duration in Experiment 2 and predicted chills duration by the machine learning model from Experiment 1.

To examine generalizability and independent validation, experiment 2 was performed with a different MRI scanner, a different participant pool, and a different stimulus set, but keeping the same model parameters derived from Experiment 1. We investigated the accuracy of predicted chills responses from Experiment 1’s machine learning model to predict actual chills responses obtained by Experiment 2 (Fig 6). Testing the success of a predictive model in an entirely separate dataset is a necessary test of population-level model generalizability [50,51]. We set *n* = 11 in Experiment 2 because it is a possible minimal significant sample to estimate the prediction accuracy from Experiment 1. That means the correlation between actual and predicted chills duration in Experiment 1 was *r* = 0.53 (Fig 2B). If the correlation strength is kept in the new dataset, *n* = 11 is a possible minimal significant sample (*r* = 0.52, *p* = .05, Pearson correlation, one side test). Because statistical studies confirmed the method for calculating the *p*-value using the beta distribution and cumulative function [52], we can calculate the minimal significant sample needed to reach significance from the number of samples.

Furthermore, to reveal whether the auditory-reward RSFC immediately before music listening is an intrinsic, stable brain network or a pre-listening dependent brain network (i.e., more trait-like or more state-like), in Experiment 2, we measured the traditional 10-min resting state before the music listening experiment. By inputting the auditory-reward RSFC score for machine learning models obtained from Experiment 1, we got prediction scores of chills-related variables both for the task and intrinsic rest. To perform a fair comparison, we calculated the same duration of auditory-reward RSFC from the pre-listening state and the intrinsic resting state by random sampling strategy (see Materials and methods).

**Fig 7:**
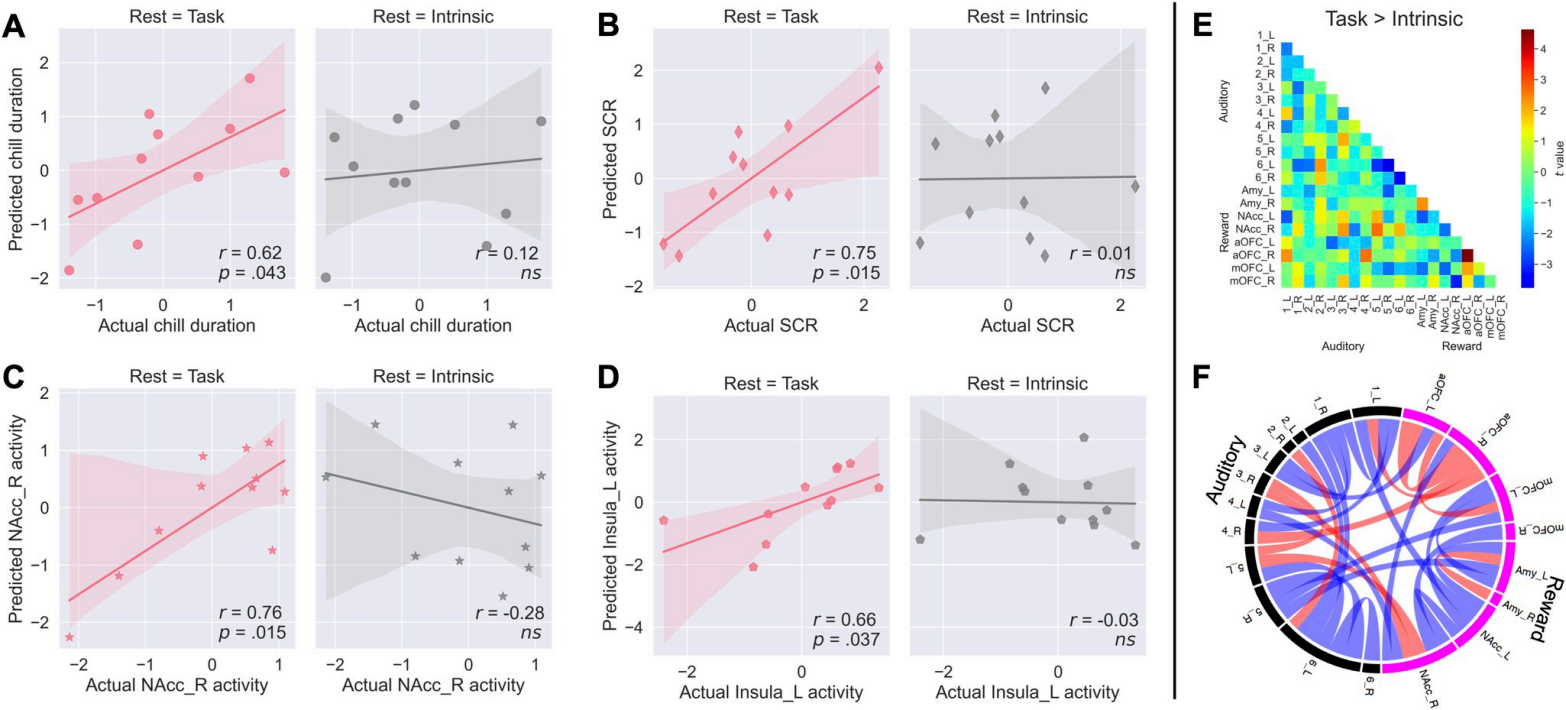
The predictive model of auditory-reward RSFC in Experiment 1 generalizes the result in the dataset of Experiment 2 for the pre-listening resting state but not for the intrinsic resting state. (**A**) Subjective chills duration, (**B**) SCR intensity, (**C**) right NAcc activity, and (**D**) left insula activity. (**E**) The difference in the auditory-reward RSFC for task-rest > intrinsic-rest. Each data point corresponds to a linear mixed model paired *t* test between the 2 conditions. (**F**) Connections passing Satterthwaite’s approximation *p* < .05 (uncorrected) are marked in red (positive) and blue (negative) in the circle for display purposes. a, anterior; Amy, amygdala; L, left; m, medial; NAcc, nucleus accumbens; OFC, orbitofrontal cortex; R, right; RSFC, resting-state functional connectivity; SCR, skin conductance response. Error bands indicating the 95% confidence interval. Underlying data and scripts are available at https://doi.org/10.17605/OSF.IO/TNRXE and in S1 Data.

This validation experiment revealed a considerable generalizability of the predictive model by the auditory-reward RSFC immediately before music listening: The correlation between actual and model-predicted responses was significant for the duration of chills (*r*_*9*_ = 0.62, *p* = .043, Fig 7A), the intensity of SCR (*r*_*9*_ = 0.75, *p* = .015, Fig 7B), right NAcc activity (*r*_*9*_ = 0.76, *p* = .015, Fig 7C), and left insula activity (*r*_*9*_ = 0.66, *p* = .037, Fig 7D) with FDR correction. In contrast, model-predicted responses using conventional intrinsic RSFC scores obtained before the experiment started did not significantly correlate with actual responses. The findings suggested that the current predictive model has generalizability only for pre-listening resting brain states. Note that the pleasure ratings obtained after the end of the excerpt were high both for self-selected (*M* = 3.41, *SD* = 0.73) and experimenter-selected (*M* = 2.86, *SD* = 0.82) music, as in the first study.

Further univariate tests with a linear mixed model showed that 2 auditory-reward RSFC networks (left aOFC-right aOFC, *β* = 0.019, SEM = 0.004, χ^2^(1) = 164, *p* = .001 and left auditory_6-right auditory_5, *β* = −0.003, SEM = 0.001, χ^2^(1) = 164, *p* = .022) significantly differed between pre-task rest and intrinsic rest (*p* < .05 with FDR correction, Fig 7E and 7F). The results support the idea that the auditory-reward network in pre-task rest differs from the intrinsic brain network and may be a state-dependent brain network.

## Discussion

In the present study, we investigated whether the auditory-reward RSFC immediately before music listening could predict emotional responses to music by machine learning. We found robust predictive ability for the auditory-reward RSFC with behavioral indicators of strong musical pleasure, i.e., chills. The auditory-reward RSFC could also robustly predict physiological arousal (SCR), NAcc, and insula activity during the chills experience. Especially, the auditory-reward RSFC in the right hemisphere predicts these chills indexes rather than the left hemisphere. As for the specific brain connection, the right auditory cortex-striatum/orbitofrontal connections were related both to behavioral responses and neural responses in NAcc and insula, whereas the auditory-amygdala connection was associated with psychophysiological arousal. Importantly, the predictive model developed for the auditory-reward RSFC from 1 sample generalized to new experimental data collected in an independent sample with different stimuli and tested with another fMRI scanner, indicating model generalizability. These results showed that the model captures relevant aspects of state-related brain activity fluctuations that predict subsequent responses.

Predicting musical rewarding experiences from pre-listening auditory-reward brain networks

We found that the duration of subjective chills experience but not neutral, pleasure, or tears responses can be predicted by the auditory-reward RSFC immediately before listening (Figs 2B and S3). Importantly, the auditory-reward network achieved the best predictive performance compared to the other 77 network combinations or whole-brain networks (Fig 2C and 2D). Musical chills represent clear and discrete events, and they provide an indicator of strong hedonic reactions to music [46], although it has a subjective and idiosyncratic nature. Our results extend the recent findings that spontaneous (but likely linked in some way to the task-related) brain states between tasks contain ample information to predict various aspects of human behavior [20–24,53] to strong pleasurable responses. Moreover, the auditory-reward network had predictability for the chills experience only when the physiological signal effect was removed (S5 Fig). As recent studies suggested the dissociation of musical pleasure and physiological arousal [54,55], we speculated that the pre-listening auditory-reward network reflects the pleasure aspect of brain activities rather than the physiological arousal aspect, i.e., we can claim from the current study that pleasure-associated pre-listening networks have predictability for subsequent listening peak pleasure experiences. Note that although FC during music listening did not predict the chill duration, the current findings did not contradict previous studies that examined auditory-reward FC during music listening. This is because these studies examined different dependent variables or conditions [9,10,25].

The current findings indicated that the auditory-reward RSFC is related to rewarding experiences such as chills, but not all ranges of musical emotion. Musical chills may be a qualitatively different emotional experience from tears and differ quantitatively from pleasure without chills. Past studies confirmed that musical chills accompany strong pleasure and arousal [4,5,37], whereas musical tears are associated with complex emotions of mixed sadness and pleasure [37,40]. We speculate a possible reason for the lack of prediction for pleasure and tears is that chills are more strongly associated with psychophysiological arousal and rewarding brain activity compared to the other responses [5,9,37,38] and may therefore be easier to detect. The sign of intense emotional arousal and reward for music may more easily emerge in the pre-listening brain networks, and the occurrence of such emotional response may depend on the biological state. Contrary to predictions, tears could not be predicted from the pre-listening brain network, possibly because tears are tied to a higher cognitive state rather than a biological state.

The psychophysiological activity of SCR, and the neural activity in the right NAcc, and left insula during the chills experience also can be predicted by the auditory-reward RSFC in a generalizable manner across experiments (Figs 3 and 7). SCR is a physiological arousal index dependent on activation of the sympathetic nervous system [56], while meta-analysis indicated consistent signal increases in the NAcc and insula in response to musical pleasure and chills [46,57,58]. Moreover, other meta-analytic results have shown that NAcc and insula show co-activation when people feel positive arousal [59] as a “neural positive arousal activity.” In accordance with this, the pre-listening auditory-reward network could determine the degree of neurophysiological positive arousal level during the chills experience. Past studies showed that the RSFC network map closely resembled the distribution of brain regions that showed increased activity during tasks [60,61]; for example, temporoparietal junction (TPJ)-linked networks predict increased activity for theory-of-mind tasks. We speculate that the auditory-reward networks could predict the NAcc/insula BOLD activities during chills responses because the functions of auditory-reward brain networks are closely associated with the brain activity for peak pleasurable experiences. In addition, the top 5 predictable brain regions in the current study (NAcc, Pallidum, pgACC, dmPFC, and insula; see Fig 3A) were included in the “liking” circuit described by Berridge and Kringelbach [62], although the predictive model of some of these regions could not be generalized in Experiment 2. The specific exchange pattern of information among auditory and reward brain systems during pre-listening may facilitate the subsequent transmission of auditory information to a liking/reward circuit [9].

The current results showed the importance of the pre-listening auditory-reward network, not simple brain activation patterns nor auditory-reward network in other periods. We assumed participants were anticipating the next music; however, anticipating the vmPFC signal and other BOLD activities did not predict the duration of chills (S1 and S6 Figs). We suggest that anticipation for the next music and musical memory/imagination are the factors that make the task-related spontaneous auditory-reward brain network. However, the predictive auditory-reward network is complex and different for each index (Fig 5), and it is difficult to determine the driving factor from the current experiment. Future studies should clarify the psychological aspect of the predictive brain network. In addition, trial-be-trial analysis of 3 different periods (Fig 2E) emphasized that a state-dependent auditory reward network before music listening is important for subsequent musical chills, but the networks are only the period connected in a predictive manner.

### Right lateralization for the predictive ability of auditory-reward brain networks

We found that the right but not left auditory-reward network significantly predicted subjective and neurophysiological responses to chills (Fig 4). These results agree with past evidence that although reward-system responses to music are mostly bilateral [57], the link between auditory and reward responses to music tends to be stronger on the right than on the left [7,9,10,25], probably because of the dominant right hemispheric lateralization for melodic and harmonic aspects of music processing [63–65]. The new contribution here is that the lateralization of brain connections emerged even before the music-listening task in the network of reward-related brain regions. The lateralization result emphasizes that the pre-connection of music processing and rewarding brain activity facilitates the evocation of musical chills. Another possibility is that musical imagery [34,35] starts before listening, and the imagination enhances the emotional response to real music.

### Predictive network specificity for subjective, physiological, and neural reward responses

The predictive network was partly different for each of the 4 substantially predicted variables. The key nodes of the subjective chill predictor included the auditory and all the reward network regions (amygdala, NAcc, and OFC) (Fig 5A). Musical chills experience would be modulated by resting auditory-reward network in general but not by any 1 specific reward region, in keeping with the idea that these structures work in tandem as part of a functional network. Past studies have emphasized the importance of the right-NAcc and auditory cortex connection during highly pleasurable music experience [9,10,25]; the current study supports this conclusion and extends it to the broad auditory-reward neural network at rest.

As a specific effect for each reward region, the pre-listening auditory-amygdala network and auditory-NAcc/OFC network were related to chills-related physiological arousal and rewarding brain activity, respectively (Fig 5B–5D). Previous research confirmed the association and causality between the amygdala and physiological arousal or NAcc/OFC and rewarding experience [25,66–68]. Moreover, the critical auditory region may be the primary sensory area for physiological arousal, whereas the right secondary auditory region was important for right-NAcc activity (S8 Fig). This anatomical dissociation suggests that arousal may be linked to lower-level acoustical features (abrupt onsets, changes in harmonicity, etc.) likely to be processed at relatively early levels of the auditory hierarchy, whereas the recruitment of the NAcc may be related to more complex cognitive operations, including predictive and mnemonic functions that require more in-depth analysis of auditory patterns and, hence, pertain to secondary auditory regions in the ventral stream of the temporal lobe [69]. The findings indicate that the physiological arousal of chills has a distinctive neural predictor compared to the rewarding neural activity of chills. Indeed, the SCR intensity during chills was not associated with NAcc activity during chills (*r*_*36*_ = −0.18). Our results therefore support the dissociation between hedonic and arousal responses of musical reward suggested by recent pharmacological studies [54,55]. Predictive networks for physiological arousal and neural reward responses could depend upon partially dissociable mechanisms.

For physiological arousal, the amygdala’s functional properties may help provide an interpretation. The amygdala receives substantial sensory information from the cortex and has a function of vigilance for salient stimuli [70]. These findings also supported the idea that connecting the primary auditory region and the amygdala may mean that amygdala can easily process auditory sensory information. Such an auditory-amygdala connection before music listening may promote chills-related physiological arousal through detecting motivationally salient auditory stimuli effectively. Past research indicated that musical chills are often experienced following sudden dynamic changes triggered by unexpected harmonies or rhythmic uncertainty [40,71,72], which would evoke physiological arousal. The pre-listening auditory-amygdala connection may facilitate the detection of these auditory cues.

The main prediction nodes of the NAcc and insula activity during the chills experience were the auditory-NAcc/OFC connection. NAcc and OFC are known to be core structures in the mesolimbic reward circuit [49,67]. Past studies also indicated that when people feel a strong pleasure from music, the functional connection between NAcc and the auditory cortex strengthens [9,10,25]. The spontaneous auditory-NAcc/OFC connection may promote neural positive arousal experiences represented by NAcc and insula intensity [59], akin to how spontaneous neural replay promotes memory task performance [53,73]. If the auditory-reward connection is already up-regulated in the pre-listening period, the connection may make it easy to engage strong neural reward for the subsequent music. In addition, as NAcc flexibly encodes the reward dimension that is currently relevant for behavior [74], NAcc rather than OFC may represent both spontaneous neural activity and relevant rewarding outcomes.

### Generalization of predictability for state sensory-reward neural connection

We used an independent-sample validation design to examine the generalizability of our predictive models. The applied validation procedure establishes the robustness of the auditory-reward predictive model, even with different brains and different music. Since we found that the generalization was possible for RSFC before music listening but not for 10-min intrinsic RSFC before the start of the experiment (Fig 7A–7D), our predictive models would be specific for the resting period immediately before music listening. We found a significant difference in several auditory-reward networks between the 2 RSFCs (Fig 7E and 7F), consistent with the suggestions of past studies that the RSFC is changeable to some degree [41,75]. These results and trial-by-trial prediction (Fig 2E) indicate that pre-listening, the auditory-reward network reflects a temporary state network rather than individual differences of the intrinsic network. If the pre-listening state plays a critical role in modulating intense emotion for music, this could be a part of the reason that music elicits different emotional responses even in the same individual at different times. It will be of interest to see if the current methodology may possibly apply to rewards from different domains besides music. For example, if the current results transfer to different modalities, the RSFC between vision or taste area and reward regions might predict strong pleasurable responses to movie viewing or food consumption. Through such examination, the general context effect of the sensory-reward brain network for pleasurable stimuli may be revealed.

Although in the present data we emphasize state-dependent response, past studies have also indicated that stable trait-related musical reward sensitivity differs across individuals and also predicts the degree of pleasurable responses [10–12,76]. It seems likely that state-dependent fluctuations of brain activity may interact with stable, trait-related brain structural and functional patterns. High-musical reward responders may more easily enter into a mode where they are likely to experience chills compared to low-musical reward responders, perhaps because the high-reward responders have a better-developed anatomical connection between the auditory and reward regions [11,12].

### Limitations

There are limitations to our study that should be kept in mind. First, one should keep in mind that musical aesthetic experiences go well beyond what can be captured with a simple button press to indicate the level of experienced pleasure. Listening to real music in a naturalistic setting involves many complex affective and cognitive reactions, some of which have been linked to brain networks that were not identified in the present analysis, such as the default mode network [77,78]. There is little doubt that a much more complex model, likely involving many more distributed brain networks [79], would be needed to explain these more subtle aspects of musically induced affective responses. Our findings concerning auditory and reward networks focus specifically on modeling liking/pleasure, and not more subtle, idiosyncratic responses or psychological states.

Second, the number of trials per participant is relatively low in the current experiment. This is because if we ask participants to select very many (30 or 40) pieces to evoke strong emotions, they would likely find it impossible to select so many pieces that could evoke strong emotions of chills/tears. However, although almost all musical reward studies used a few trials to maximize reward responses [4,5,12,80], the results have been consistent across studies, as shown in a recent meta-analysis [57].

Third, pre-registration has been popular in the field of predictive modeling research and should help to reduce analytic flexibility in neuroimaging data. Here, only the single model derived from Experiment 1 was tested on the generalization datasets from Experiment 2, without any further modification. Pre-registration in future studies could further facilitate analytic rigor. Indeed, having demonstrated the validity and out-of-sample generalization of the model, it can now serve for pre-registration of future studies that explore its utility in different contexts.

In addition, we must be cautious about the precise anatomical location of RSFC nodes within the brainstem, because the size of the ROIs in the current study was relatively large (8 mm spheres) compared to the size of some subcortical structures.

## Conclusions

Our results demonstrate that the connectivity of the auditory-reward network immediately before music listening can robustly predict subjective, physiological, and neurobiological aspects of musical chills. Specifically, the right secondary auditory-NAcc/OFC connections were related to neural positive arousal responses, whereas the peri-primary auditory-amygdala connections were associated with physiological arousal. The brain decoder based on the auditory-reward network could predict chills experience in an independent dataset from the pre-listening, but this was not the case for the intrinsic resting brain network. Taken together, the findings suggest that the pre-listening auditory-reward network contains information about the responses to strong musical rewards before experiencing them. A key factor of why music becomes a strong reward may be the pre-listening state, which efficiently exchanges information between auditory and reward systems. For example, during a live concert, when you experience peak pleasure in music, it is thought to have a critical role that the auditory-reward brain network immediately before listening exhibits a specific connection by anticipation and other cognitive states. The highly rewarding music experience may start even before listening to music. If this is a general rule for our reward experience (not only music but also, for example, movies, sports, and food), the rewarding experience could depend on the pre-connection state between sensory and reward brain regions, which may reflect the anticipation of how the next sensory experience will unfold in a pleasurable manner.

## Materials and methods

### Ethics statement

The local ethics board granted ethical approval for the study (the human ethics research committee of the National Institute of Information and Communications Technology (NICT), P195154/P195654). The experimental protocol was conducted in accordance with the Declaration of Helsinki. All participants provided written informed consent.

### Participants

In the sampling phase, we recruited participants through web advertisement. Using a web-based survey, we asked them to complete 2 questionnaires to assess the frequency at which they experienced chills and tears while listening to music. The prevalence of the intense emotional responses to music was assessed based on the answers to the Barcelona Music Reward Questionnaire (BMRQ) [76] and Aesthetic Experience Scale in Music (AES-M) [12]. In Experiment 1, 43 participants who sometimes experienced chills and tears were recruited. Four participants were removed because they did not report any chills or tears responses during the experiment. One participant was removed due to having an abnormality of autonomic nervous system activity. In Experiment 2, independent 12 participants were recruited. One participant was removed due to misunderstanding online emotion ratings (many button presses under 1 s). Final sample was 38 in Experiment 1 (14 women; age: *M* = 21.8, *SD* = 1.4; BMRQ: *M* = 77.6, *SD* = 10.0, *Max* = 96, *Min* = 54; AES-M: *M* = 50.9, *SD* = 10.9, *Max* = 74, *Min* = 20) and 11 in Experiment 2 (2 women; age: *M* = 21.9, *SD* = 1.6; BMRQ: *M* = 83.3, *SD* = 10.4, *Max* = 99, *Min* = 69; AES-M: *M* = 67.8, *SD* = 17.1, *Max* = 96, *Min* = 41). The mean score of Experiment 1 was near to the score of the average musical reward sensitivity group [10], then the current main results would apply average university population. From these questionnaire scores, we confirmed that the participants did not include strong musical anhedonia [10].

For Experiment 1, a target sample size was determined according to similar previous studies predicting behavior from RSFC, *N* = 35 [81], and brain fluctuations immediately before the task, *N* = 43 [24]. In addition, we verified that the sample size was appropriate using learning curve analysis (see S2 Fig) (sample size for Experiment 2 is explained in Results). Participants were instructed to abstain from alcoholic beverages the night before the experiment and avoid coffee or cigarettes for 3 hours before the experiment. Participants were compensated for their participation in the study.

### Experimental procedure

Before the experiment, participants were instructed to select 4 songs, i.e., 2 pieces of strong pleasurable chill- and 2 tear-evoking songs (S2 and S3 Tables). To keep the music style uniform across participants, participants were asked to select music stimuli from pop/rock genres. In Experiment 1, the experimenter additionally selected 4 popular songs based on the previous year’s hit chart in Japan (S1 Table). We avoided songs that would be expected to be nonpreferred to the listeners. The songs most commonly included vocals, guitar, bass, and drums as similar as self-selected songs. The mean length of self-selected music was 257 s (*SD* = 24.4), whereas experimenter-selected music was 240 s (*SD* = 48). In Experiment 2, to generalize the machine learning model for different experimental paradigms, experimenter-selected songs were used from the self-selected music of other participants. Specifically, Participant A’s 4 self-selected pieces were assigned to Participant B, and Participant B’s 4 self-selected pieces were assigned to Participant A [4,5].

In the fMRI experiment, participants first engaged in a training session to familiarize themselves with the experimental procedure. After that, participant performed 2 fMRI sessions. Each session consisted of 1 run in which the participants listened to 2 self-selected songs and 2 experimenter-selected songs. The order of presentation of songs was pseudo-counterbalanced, which mixed between self-selected and experimenter-selected songs to enhance the uncertainty of emerging songs across trials (see S1 Fig) and not running out the self-selected music first, which may weaken the expectations about the next music. Each trial started with an instruction lasting 5 s, followed by a musical excerpt with a maximum duration of 270 s in Experiment 1 (*M* and *SD*; see above) or a maximum duration of 360 s in Experiment 2 (*M* = 281.1 s, *SD* = 12.6). Since the instruction provided simple information such as “Next music coming soon,” the participants could not infer the next music from the instruction. In addition, the time interval between each musical piece was constant.

The participants had to indicate, in real time, their emotional responses while listening to the music by pressing 1 of 4 different buttons on an MRI-compatible response pad (1 = neutral, 2 = pleasure, 3(4) = chill, 4(3) = tear) in their right hand. Chills were defined as “goosebumps” or “shivers down the spine,” and tears were referred to as “weeping” or “a lump in the throat” [4,5,37,38]. These responses are considered physiological reactions, but we call emotions in the current research to avoid the confusion of physiological measures (HR, RR, and SCR). The position of the chill and tear buttons was counterbalanced across participants to avoid ordering bias for these responses. The participants were instructed, “Please always press one of the four buttons and report your feelings online. Basically, you can press one of the four buttons. However, chills and tears may co-occur, so chills and tears can be pressed at the same time.” The participants were also instructed to hold down the button as long as they experienced the corresponding degree of emotion. Button signals were recorded at a frequency of 100 Hz. Note that the duration of the excerpt was not correlated with the duration of any 4 emotional responses (*p*s > .45). At the end of each excerpt, the participants were asked to rate 16 items (from 1 = not at all to 4 = very strong) they felt in response to the musical excerpt. The items included pleasure, chills, and tears (we do not report all the rating items here and plan to report them in another study). Each item was required to respond within 5 s. After the experiment, participants were asked to answer the familiarity (from 1 = unfamiliar to 4 = very familiar) for the experimenter-selected music.

To measure RSFC immediately before music listening, after each song was played, the participants relaxed for 50 s in Experiment 1 and 40 s in Experiment 2. We intend 80 s for 16 rating items to reduce the carry-over effect of music listening for the RSFC. To avoid the remaining effect of the rating task, a further 10 s was removed from the examination of task rest (i.e., task rest for Experiment 1 was 40 s and Experiment 2 was 30 s, but it seems RSFC change was a continuous rather than radical during the removed period; see S10 Fig).

### Quantifying behavioral measurement

The onset time and duration were recorded for every 4 emotional responses. Button presses of less than 1 s were not analyzed to avoid miss pressing. We removed button presses that were the same as the previous buttons and concatenated the duration of these 2 button presses. Since we asked the participant to press 1 button every time during music listening, we assumed the 2 presses were sustained when participants repeatedly pushed the same button. After that, we totaled the duration of each of the 4 types of buttons in 1 song. For the main analysis, we used the average of each 4 durations for all songs for each individual. The grand average duration of 4 emotional responses is as follows: neutral (*M* = 41.5 s, *SD* = 28.2), pleasure (*M* = 127.2 s, *SD* = 34.5), chill (*M* = 50.7 s, *SD* = 35.1), tear (*M* = 37.5 s, *SD* = 24.2) (for more detail, see S5 Table). In addition, the average familiarity score of the experimenter-selected songs was 2.95 (*SD* = 0.63).

### Brain image and physiological acquisition

In Experiment 1, data were acquired using a 3T Siemens Trio scanner equipped with a 32-channel head coil. Music stimuli were presented via Sensimetrics MRI-compatible insert earphones (S-14) at a comfortable volume. We recorded 2 experimental runs corresponding to the 2 blocks of trials in the main experimental task. Functional volumes were acquired using a T2*-weighted gradient echo, EPI sequence (62 interleaved slices, gap: 0.3 mm, voxel size: 3 × 3 × 3 mm, matrix size: 64 × 64, FOV: 192 mm, TE: 30 ms, TR: 2,000 ms, flip angle: 75°, multiband factor: 2). Additionally, a high-resolution anatomical volume was acquired at the mid-rest of the experimental session using a T1-weighted sequence (208 slices, no gap, voxel size: 1 × 1 × 1 mm, matrix size: 256 × 256, FOV: 256 mm, TE: 3.37 ms, TR: 1,900 ms, flip angle: 9°), which served as anatomical reference for the functional scans.

In Experiment 2, neuroimaging data were acquired on a 3T Siemens Prisma scanner with a 64-channel head coil. Both functional and anatomical volumes were acquired with the same parameters as Experiment 1. Before the experiment, a resting-state fMRI session of 10 min (600 volumes) was followed with the parameters of T2*-weighted functional image acquisition (72 interleaved slices, gap: 0.16 mm, voxel size: 2 × 2 × 2 mm, matrix size: 100 × 100 mm, FOV: 200 mm, TE: 30 ms, TR: 1,000 ms, flip angle: 60°, multiband factor: 6). The parameter acquisition was determined for a different purpose.

Concurrent with functional imaging, physiological recordings were also acquired using an MR-compatible BIOPAC MP150 polygraph (BIOPAC Systems, USA) for Experiments 1 and 2. Cardiovascular, respiration, and electrodermal signals were obtained with a sampling frequency of 1,000 Hz. Respiratory activity was assessed by a strain gauge transducer incorporated in a belt tied around the chest. To get a cardiac signal from a photo plethysmogram, the optical pulse sensor was attached to the proximal phalanx of the pinky finger of the participant’s left hand. Skin conductance was measured continuously with Ag/AgCl electrodes placed at the index and middle fingers of the left hand.

### Brain image and physiological data pre-processing

Functional MRI data were pre-processed using Statistical Parametric Mapping software (SPM12; http://www.fil.ion.ucl.ac.uk/spm). The analysis used a task resting state (10 + 40 s in Experiment 1 and 10 + 30 s in Experiment 2), instruction 5 s, and a piece of music as a single block. Therefore, 8 BOLD time series were analyzed separately for each participant. Since the length of the music varied (see above), the length of each block also varied. Distortion correction was applied using field maps. Functional images were realigned to the mean image of the series, slice-time corrected, motion corrected, co-registered to the structural image, normalized to MNI space, and spatially smoothed with a 6-mm FWHM Gaussian kernel. Potential outlier scans were identified from the resulting subject-motion estimates as well as from BOLD signal indicators using default thresholds in CONN toolbox (https://web.conn-toolbox.org/home) preprocessing pipeline (5 standard deviations above the mean in global BOLD signal change, or framewise displacement values above 0.9 mm).

Afterward, using CONN toolbox default settings [82], the common nuisance variables were regressed out, including the subject-specific white matter mask and the cerebrospinal fluid signal mask (5 PCA parameters, CompCor), 12 movement regressors (6 head motion parameters and their first-order temporal derivatives), and scrubbing defined by outlier scans. Physiological noise regressors were also included as the nuisance variables. Tapas PhysIO Toolbox [83] was used to calculate HR- and RR-related nuisance variables, including cardiac response function, respiration function, and RETROICOR at the same temporal resolution as the fMRI time series. Since the previous study indicated the relationship between resting physiological arousal and musical chills [84] and HR/RR activities could be the critical noise for RSFC [44], removing the physiological noise is important to know the link between resting brain state and musical emotion. The linear trends of time courses were removed, and band-pass filtering (0.008 to 0.09 Hz) was applied to the time series of each voxel to reduce the effect of low-frequency drifts and high-frequency physiological noise.

### RSFC feature extraction

ROIs were defined using a functional brain atlas, which was derived from resting-state fMRI data based on InfoMap and a winner-take-all parcellation algorithm [42,45]. The atlas includes 288 ROIs spanning the whole brain, including the cerebellum and brainstem. The spherical ROIs were diameter = 8 mm. Each ROI was assigned to each 13 functional network communities (see Fig 2A). For each participant, a time course was computed for each ROI by averaging the BOLD signal of its constituent voxels at each time point. Basically, the 40-s time-series BOLD signal was used for each 8 task-rest period for each participant. However, we subsequently changed the time-series duration according to the analysis plan such as moving window analysis in Fig 2F.

Functional connectivity between each pair of ROIs was calculated as the partial correlation (Pearson’s r) between the time courses of each pair of ROIs, yielding 1 correlation matrix per task rest per participant. Specifically, we calculated the correlation between the time series of 2 ROIs after adjusting for the time series of all other 286 ROIs in the whole brain. Partial rather than simple correlations were used to rule out indirect connectivity effect [85]. Fisher’s r-to-z transformation was then implemented to improve the normality of the correlation coefficients, resulting in 288 × 288 symmetric connectivity matrices representing the set of connections in each RSFC profile immediately before music listening. After that, the upper triangle of these matrices was used as the feature space for machine learning–based predictive modelling. Note that 4 trials of RSFC from different individuals were removed because Tapas PhysIO Toolbox cannot compute physiological denoising variables.

For predictive modelling, we used a network (includes several ROIs) seed-based functional connectivity to examine our auditory-reward network hypothesis and another possibility related to the auditory network. We set each 12 auditory seed networks as a feature (e.g., Auditory-Reward, Auditory-Default mode). We calculated the partial correlation matrix 12 (6-left and right) auditory ROI and several ROI in other networks (e.g., Reward: 8, Default mode: 65). We also examined whole-brain functional connectivity according to the distribution model of emotional brain [79,86]. In addition, we exploratory investigated the other possible 66 combinations of 2 networks (e.g., Visual-Reward, Salience-FrontoParietal).

### Predictive modelling of emotional responses using RSFC and statistical analysis

We used LASSO regression to decode each of the duration of emotional responses (neutral, pleasure, chill, tear) from RSFC immediately before music listening. The analysis was conducted using scikit-learn 1.0.2 in Python 3.8.13. In the main analysis, we set features as the averaged FC from each 8 task rest. The target (dependent variable) was the average of each of the 4 emotion durations across 8 pieces.

First, RSFC features are standard scaled. Next, a LASSO decoder was trained, wherein an L1 regularization parameter λ was used for shrinkage and tuned to control overfitting. After averaging the 8 RSFCs per participant, nested LOPOCV was applied, with outer LOPOCV estimating the model’s generalizability and the inner LOPOCV to determine the optimal parameter λ. In the outer LOPOCV, 37 participants were used as the training set, with the remaining used as the testing set. Based on the optimal λ (see next paragraph), a model was trained using all participants in the training set, and the model was then used to predict the outcome of a participant in the testing set. For all 38 participants, the above procedure was repeated.

Within each loop of the outer LOPOCV, the inner LOPOCVs were applied to determine the optimal λ. The training set for each loop of the inner LOPOCV was further partitioned into 37 participants, similar to the outer loop. Under a given λ in the range [0.001, 0.01, 0.1, 1, 10, 100, 1,000], 36 participants were selected to train the model, and the remaining 1 participant was used to test the model. This procedure was repeated 37 times to ensure that each participant was used once as the testing dataset, thereby resulting in a total of 37 inner LOPOCV loops. For each λ value, accuracy between the actual and predicted outcomes was calculated for each inner LOPOCV loop and averaged across the 37 inner loops. The mean accuracy was defined as the inner prediction accuracy, and λ with the highest inner prediction accuracy was selected as the optimal λ for the outer LOPOCV.

Parametric and non-parametric statistical tests were used to evaluate the decoding results for the duration of musical emotion. To evaluate if the prediction performance was significantly better than would be expected by chance, we performed a correlation test. Furthermore, we conducted a permutation test only for significantly correlated variables to reduce computational time. As for the permutation test, for repeated random LOPOCV, the outer LOPOCV was repeated 10,000 times, but each time the duration of musical emotion across the training data was permuted without replacement. We calculated the *p*-value of obtaining a mean correlation in the null distributions equal to or higher than the true correlation from the analyses. FDR correction (Benjamini–Hochberg) was applied to correct multiple comparisons of the *p*-value.

### Trial-by-trial predictive modelling using the 3 periods of the auditory-reward network

To complement the result of the main analysis, we investigated whether trial-by-trial RSFC can predict the subjective duration of chills. We attempted to predict the duration of musical chills from the preceding auditory-reward RSFC as like LOPOCV analysis. We also attempted to predict the ongoing duration of chills from the auditory-reward FC during music listening because task FC may have a higher predictive ability of behavior than RSFC [87,88]. In addition, we tried to predict the duration of chills from the following auditory-reward RSFC. The chills experienced for the preceding music listening may influence the auditory-reward RSFC. If this is true, the auditory-reward RSFC should predict a backward chills experience.

Like the LOPOCV-LASSO model, we construct a nested LOTOCV-LASSO model. First, we removed 2 outlier trials above 3 standard deviations, resulting in 298 trials being analyzed. Even if we did not remove outliers, the analysis showed the same results. In the inner loop, 296 trials were selected to train the model, and the remaining 1 trial was used to test the model to determine optimal λ. This procedure was repeated 297 times to ensure that each trial was used once as the testing dataset, resulting in 297 inner LOTOCV loops. For each λ value, accuracy between the actual and predicted outcomes was calculated for each inner LOTOCV loop and averaged across the 297 inner loops. In the outer LOTOCV, 297 trials were used as the training set, with the remaining one used as the testing set. Using the optimal λ, the model was trained using all trials in the training set, and the model was used to predict the outcome of a trial in the testing set, repeating for all 298 trials. Note that because we did not have the post-RSFC for the last trial for every 2 sessions, we used 222 trials to make the LOTOCV-LASSO model that predicts the backward chills experience. To test the significant prediction ability of chills experience, we conducted a correlation test and 1,000 permutation tests to investigate whether actual chills duration and predicted chills duration have a statistically significant relationship.

### Validation of RSFC predictive modelling

The prediction results of the auditory-reward network both for the LOPOCV- and LOTOCV-LASSO models were further validated with a range of different RSFC time windows. We set RSFC time windows by decreasing 1 TR. Because prior evidence suggests that 22.5-s functional connectivity can distinguish distinct cognitive tasks [22], we set RSFC time windows ranging from 20 to 40 s (20, 22, …, 40 s). Furthermore, using the minimum significant 26-s time windows (see Fig 2F-left), the sliding window analysis was performed over successive 26-s RSFC windows (24 s overlapping) from 0 to 40 s (0 to 26, 2 to 28, …, 14 to 40 s). We conducted a correlation test on actual chill duration and predicted chill duration.

### Physiological and neural intensity of the emotional chills

To investigate whether RSFC can predict not only subjective duration but also physiological arousal and neural activation during musical chills, we examined the intensity of physiological and neural responses while participants experience chills.

HR and RR were quantified after band-pass filtering raw signal (HR: low-pass 35 Hz, high-pass 1 Hz; RR: low-pass 1 Hz, high-pass 0.05 Hz). HR was calculated by the inverse instantaneous inter-beat intervals (in ms) from the photo plethysmogram using a peak detection algorithm implemented by SciPy 1.9.0 to detect successive R-waves. Careful visual confirmation of the electrocardiogram ensured that the automatic R-wave detection procedure had been performed correctly. We then performed cubic spline interpolation implemented by SciPy 1.9.0 within the 2 successive HR values to obtain 10 Hz time-series HR data. RR was also calculated by the peak detection algorithm and cubic spline interpolation. SCR data were analyzed using Ledalab (Version 3.4.9, MATLAB). Data were low-pass filtered (1 Hz) and downsampled to 10 Hz. Continuous decomposition analysis (CDA) was performed to extract the phasic SCRs from the electrodermal activity [56]. CDA yields the phasic driver underlying skin conductance data as a continuous measure of SCR, which would be robust to common artifacts. We examined only SCR responses above 0.05 μS, which indicates an unambiguous increase in skin conductance [84]. SCR scores were transformed by log function to modify distribution bias [56].

We calculated HR, RR, and SCR scores during the chills experience after synchronizing these scores to subjective responses. For every subjective chill experience, the average physiological response for the 1-s time window before the onset was calculated as a baseline. Any score exceeding 3-point standard deviations from the mean was removed as an outlier. Finally, the sum of respective measures (HR, RR, SCR) over the duration of each button press minus the 1 s before the onset baseline as the dependent variable.

Neural responses during the chills experience were quantified by the BOLD signal from each of 140 ROIs from automated anatomical labelling (AAL) 3.1 [47], excluding cerebellum regions. The pre-processing was the same as RSFC data except for filtering: The time series BOLD signals were high-pass filtered with a cutoff of 0.008 Hz (125 s). To avoid removing task-related neural activity, the low-pass filter was not used for the BOLD signal. Percent signal change was calculated relative to the average of 2 volumes, which were included before and after 1 volume on chills onset, to correct for differences in starting baseline. Copied time courses are shifted by 4 s to account for the hemodynamic delay of the fMRI signal. Data that deviate from the average by more than 3 standard deviations were removed for quality assurance [59]. We investigated the sum of percent signal change during chill response as each 140 neural activity scores. We conducted correlation and 10,000 permutation tests to examine the relationship between actual physiological and neural activities and predicted these activities. FDR correction was applied to correct multiple comparisons.

### Asymmetry analysis of the auditory-reward RSFC

We tested for rightward asymmetry of emotional responses [7,9,10,25] by performing a machine learning prediction on the difference between the subjective duration, SCR intensity, right-NAcc activity, and left insula activity of chills in the right versus left ROIs, using the 40-s auditory-reward RSFC. We used 45 connections for a 10 × 10 partial correlation matrix (6 auditory and 4 reward ROIs) from each hemisphere to make the LOPOCV-LASSO model. To test the hypothesis that the right but not left hemisphere relates to neuropsychological responses to chills, we conducted correlation and 10,000 permutation tests on actual chills experience and predicted chills experience. FDR correction was applied to correct multiple comparisons. Furthermore, to test the difference in prediction accuracy (Pearson’s r) between the right and left hemispheres, we performed 10,000 bootstrap tests [89].

### Generalization of predictive model

In Experiment 2, participants were presented with 8 songs (4 self-selected and 4 experimenter-selected). The target was set as the mean duration of subjective chills responses for 8 songs for each participant. The single auditory-reward RSFC immediately before music listening (task rest) was calculated and then the within-participant RSFC with the 8 songs was averaged, which resulted in 1 RSFC for each participant. The duration of RSFC varied between 6 and 40 s. By inputting the auditory-reward RSFC score for the machine learning model from Experiment 1, we got the predicted score of subjective, physiological, and neural aspects of the chills experience for each RSFC duration. Furthermore, we calculated the auditory-reward RSFC from the traditional 10-min resting state (intrinsic rest) to examine whether the intrinsic brain network is different from RSFC immediately before music listening. To compare equal conditions for 2 types of RSFC, we sampled 8 short-time (each 18 s; see below) BOLD signals from 10 min of resting brain state. To avoid biased sampling, random sampling was performed for the 10-min resting state and repeated 10,000 times. The auditory-reward RSFC scores averaged 10,000 samplings were entered into the machine learning model from Experiment 1 and got the predicted scores for the intrinsic resting brain state. Since the prediction accuracy summing subjective duration, SCR, right-NAcc, and left insula was highest when we analyzed the 18-s auditory-reward RSFC (see S10 Fig), we decided to use 18 s both for the task and intrinsic rest. The evaluation of prediction accuracy for each task and intrinsic rest was performed by a one-sided Pearson correlation test. FDR correction was applied to correct multiple comparisons.

In addition, in a complementary analysis to the machine learning approach, we examined a univariate analysis of the difference in the 18-s auditory-reward RSFC scores between task and intrinsic rest. Using each of the 8 auditory-reward network pairs for each participant, we performed a linear mixed model with a significance set at Satterthwaite’s approximation by lme4 [90].

## Supporting information

S1 Fig Anticipation-related brain responses for next music. (**A**) Anticipation effect emerged in vmPFC during rest immediately before music listening. vmPFC ROI was merged bilateral “Rectus” and “OFCmed” in AAL3 [47]. Linear mixed model analysis showed a significant difference between max (36 s) and min (24 s) points displayed as star marks (Satterthwaite’s approximation *p* = .020) [90]. Note that the anticipation signal (the difference score between 36 s and 24 s in vmPFC) did not significantly correlate with the duration of chills (*r*_36_ = −.19, *p* = .26). (**B**) The music order of Experiment 1 was pseudo-randomized to evoke unpredictability and anticipation in participants. ROI, region of interest; vmPFC, ventromedial prefrontal cortex.
(TIF)

S2 Fig Learning curve analysis. The results of learning curve analysis for LOPOCV analysis by auditory-reward RSFC in Experiment 1. After training set size beyond 50% (*n* = 19), classification accuracy shows convergent and stability. LOPOCV, leave-one-participant-out cross-validation; RSFC, resting state functional connectivity.
(TIF)

S3 Fig Machine learning prediction for the duration of neutral, pleasure, and tears. Performance of auditory-seed predictive model for the subjective duration of neutral, pleasure, and tear. Each dot shows the results of LOPOCV prediction using the different combinations of auditory and other networks plus the whole brain network. LOPOCV, leave-one-participant-out cross-validation.
(TIF)

S4 Fig Predictive performance of the auditory-reward model by the LOTOCV. (**A**) The correlation coefficients were calculated by each music trial. RSFC-preceding, mean *r*_36_ = 0.28, *SD* = 0.13, FC-music listening, mean *r*_36_ = 0.09, *SD* = 0.19, RSFC-following, mean *r*_36_ = 0.08, *SD* = 0.14. Predictive performance varied between trials, but it was difficult to find a specific tendency (e.g., better performance in the first half and worse performance in the second half). Note that for the prediction for each song, machine learning was not performed with data about individual songs. From the machine learning results using data from all trials, we extracted the predicted and measured scores for each song and examined the correlations. (**B**) The correlation coefficients were calculated by each self- and experimenter-selected music condition. RSFC-preceding, self: *r*_36_ = 0.29, experimenter: *r*_36_ = 0.25, FC-music listening, self: *r*_36_ = 0.10, experimenter: *r*_36_ = 0.05, RSFC-following, self: *r*_36_ = 0.10, experimenter: *r*_36_ = 0.06. Since the correlation coefficient between actual chills and predicted chills was very close for the self- and experimenter-selected music condition, the trial-by-trial machine learning model captured the overall tendency of chill responses regardless of the 2 music types. Each dot represents each trial. Error bands indicate the 95% confidence interval. Exp, experimenter; FC, functional connectivity; LOTOCV, leave-one-trial-out cross-validation; RSFC, resting state functional connectivity.
(TIF)

S5 Fig Performance of the auditory-reward predictive model for the duration of chill without removing physiological noise. The pre-listening auditory-reward network, as well as right NAcc and left insula activity during the chills experience, was calculated from a time series BOLD signal without the physiological covariates derived from [83] removed. Each dot shows the results of LOPOCV prediction using the auditory-reward brain network. LOPOCV, leave-one-participant-out cross-validation; NAcc, nucleus accumbens.
(TIF)

S6 Fig BOLD activity patterns during 40-s pre-listening rest in 2 separate epochs. (**A**) Results of a whole-brain comparison between 2 epochs. The comparison of the first half to the second half revealed greater activity in the bilateral auditory cortical regions (FWE, *p* < .05). (**B**) The reverse contrast illustrated greater activity on the vmPFC/NAcc, hypothalamus, and vision regions (uncorrected, *p* < .005 for display purposes). (**C**) Time-dependent increasing or decreasing tendencies of BOLD activity (each dot shows each participant). (**D**) Performance of BOLD activity patterns predictive modeling for chills-related variables using LOPOCV. a, anterior; Amy, amygdala; L, left; LOPOCV, leave-one-participant-out cross-validation; m, medial; NAcc, nucleus accumbens; OFC, orbitofrontal cortex; R, right; SCR, skin conductance response; vmPFC, ventromedial prefrontal cortex. See also S1 Text and S4 Table.
(TIF)

S7 Fig Performance of auditory-reward predictive model for the chills-related pgACC, dmPFC, and Pallidum activations. (**A**, **B**, **C**) Prediction accuracy for pgACC, dmPFC, and Pallidum activities during chills experience. The actual versus predicted neural activity by LOPOCV is shown in the plots. (**D**, **E**, **F**) The positive and negative predictive weight of auditory-reward RSFC for pgACC, dmPFC, and Pallidum. dmPFC, dorsomedial prefrontal cortex; L, left; LOPOCV, leave-one-participant-out cross-validation; pgACC, pregenual anterior cingulate cortex; R, right. Note that we show left pgACC, dmPFC, and Pallidum prediction plots in the Supporting information since these predictions did not generalize in Experiment 2 (see also S10 Fig).
(TIF)

S8 Fig Performance of primary auditory-reward and secondary auditory-reward predictive model for the chills-related SCR and right NAcc. (**A**) The early (red) and higher-order (brown) auditory regions from HCP [48] and auditory network (white) from [44]. Using the early or higher-order auditory ROI from [48] and reward network from [44], we performed LOPOCV analysis. (**B**) Prediction accuracy for (**B**) SCR intensities and (**C**) NAcc activities during chills experience as a function of bilateral, left, and right brain networks and the 2 types of auditory ROI. HCP, human connectome project; L, left; LR, left and right; LOPOCV, leave-one-participant-out cross-validation; NAcc, nucleus accumbens; R, right; ROI, region of interest; SCR, skin conductance response.
(TIF)

S9 Fig The positive and negative predictive weight of right auditory-reward RSFC. (A) Subjective duration, (B) SCR intensity, and (C) right NAcc activity of chills. Nodes are sized and colored as in Fig 5. Predictive weights showed a similar tendency as the bilateral hemisphere weight. Auditory-amygdala connection showed a higher weight for SCR, whereas the auditory-NAcc/OFC connection showed a higher weight for right NAcc. a, anterior; Amy, amygdala; L, left; NAcc, nucleus accumbens; m, medial; OFC, orbitofrontal cortex; R, right; RSFC, resting state functional connectivity; SCR, skin conductance response.
(TIF)

S10 Fig Prediction accuracy for subjective, physiological, and neural activities of chills experience as a function of RSFC duration in Experiment 2. For each chills-related variable, we computed the accuracy of predicted responses from Experiment 1’s machine learning model to predict actual responses obtained by Experiment 2. dmPFC, dorsomedial prefrontal cortex; L, left; NAcc, nucleus accumbens; pgACC, pregenual anterior cingulate cortex; R, right; RSFC, resting state functional connectivity; SCR, skin conductance response.
(TIF)

S1 Table Information of experimenter-selected music in Experiment1. Fixed 4 experimenter-selected songs for Experiment 1. The selection was based on the 5-download rankings in Japan in 2017.
(HTML)

S2 Table Information of self-selected music in Experiment 1. Songs were selected for Experiment 1 in this study. Each participant selected 4 pieces of music.
(HTML)

S3 Table Information of self-selected music in Experiment 2. Songs were selected for Experiment 2 in this study. Each participant selected 4 pieces of music.
(HTML)

S4 Table Whole-brain GLM for task-rest in Experiment 1. Regions of a whole-brain univariate GLM analysis showing significantly different BOLD responses between the first and second half epochs. ^a^All regions are statistically significant at *p* < .05 after whole-brain correction for family-wise error multiple comparisons. ^b^Voxels at a voxel-wise significance threshold of uncorrected *p* < .001. ^c^This table reports all clusters containing ≧4 voxels, and cluster size indicates the number of voxels at an isotropic resolution of 3 mm. The coordinates refer to MNI space.
(DOCX)

S5 Table The average duration (SD) of each 4 emotional response for each 3-music type. (DOCX)

S1 Text Analysis method for BOLD activity patterns during 40-s pre-listening rest in 2 separate epochs. (DOCX)

S1 Data Data underlying the plots in Figs 2–5 and 7. (XLSX)

## Abbreviations

AAL: automated anatomical labelling
AES-M: Aesthetic Experience Scale in Music
BMRQ: Barcelona Music Reward Questionnaire
CDA: continuous decomposition analysis
FDR: false discovery rate
fMRI: functional MRI
HR: heart rate
LASSO: least absolute shrinkage and selection operator
LOPOCV: leave-one-participant-out cross-validation
LOTOCV: leave-one-trial-out cross-validation
NAcc: nucleus accumbens
ROI: region of interest
RSFC: resting-state functional connectivity
RR: respiration rate
SCR: skin conductance response
TPJ: temporoparietal junction
vmPFC: ventromedial prefrontal cortex
