# Supplementary figures and images for: State-dependent connectivity in auditory-reward networks predicts peak pleasure experiences to music
Source: PLoS Biol. 2024 Aug 12;22(8):e3002732. doi: 10.1371/journal.pbio.3002732 (PMC11318860; doi:10.1371/journal.pbio.3002732)

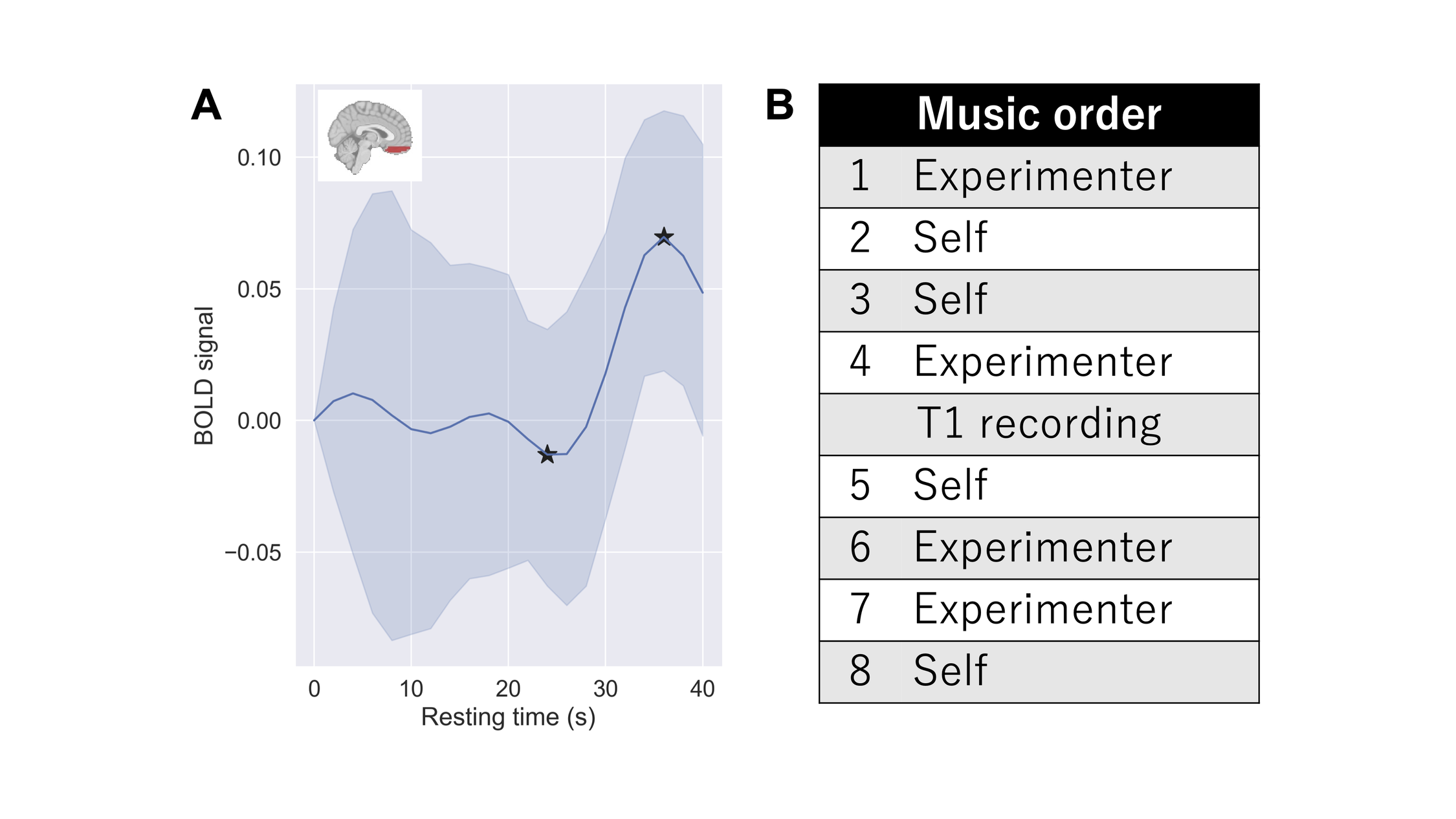

Supplement: S1 Fig — (A) Anticipation effect emerged in vmPFC during rest immediately before music listening. vmPFC ROI was merged bilateral “Rectus” and “OFCmed” in AAL3 [47]. Linear mixed model analysis showed a significant difference between max (36 s) and min (24 s) points displayed as star marks (Satterthwaite’s approximation p = .020) [90]. Note that the anticipation signal (the difference score between 36 s and 24 s in vmPFC) did not significantly correlate with the duration of chills (r36 = −.19, p = .26). (B) The music order of Experiment 1 was pseudo-randomized to evoke unpredictability and anticipation in participants. ROI, region of interest; vmPFC, ventromedial prefrontal cortex. (TIF) [file pbio.3002732.s001.tif]

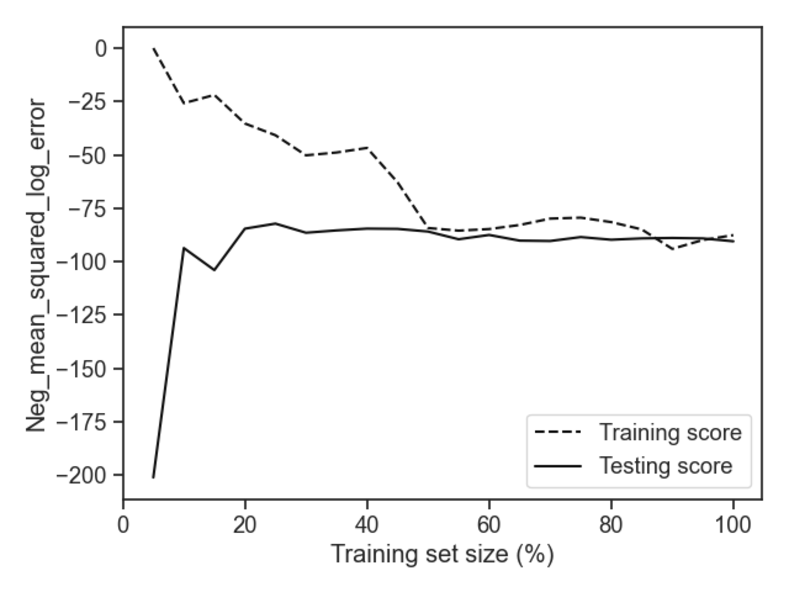

Supplement: S2 Fig — The results of learning curve analysis for LOPOCV analysis by auditory-reward RSFC in Experiment 1. After training set size beyond 50% (n = 19), classification accuracy shows convergent and stability. LOPOCV, leave-one-participant-out cross-validation; RSFC, resting state functional connectivity. (TIF) [file pbio.3002732.s002.tif]

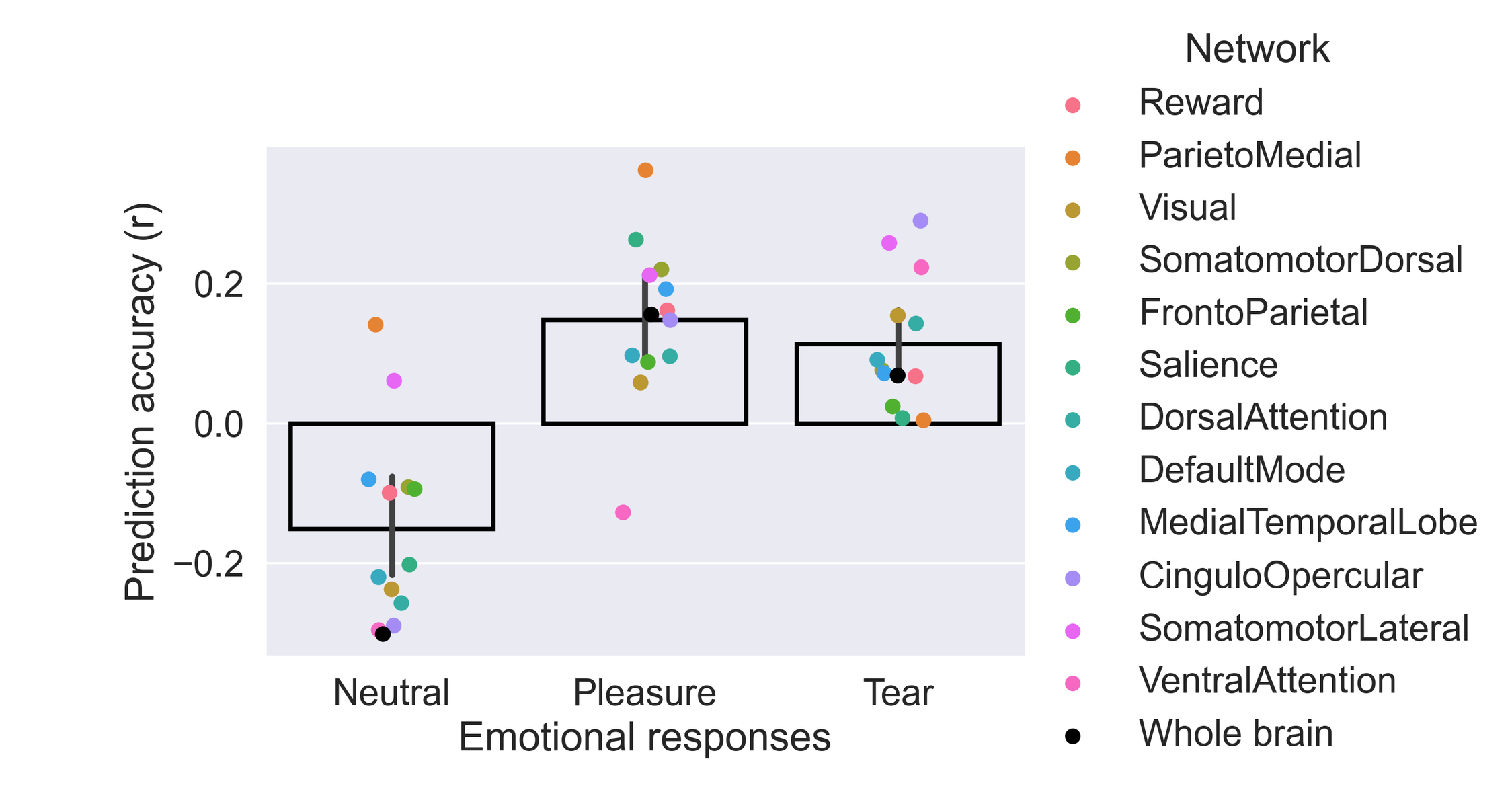

Supplement: S3 Fig — Performance of auditory-seed predictive model for the subjective duration of neutral, pleasure, and tear. Each dot shows the results of LOPOCV prediction using the different combinations of auditory and other networks plus the whole brain network. LOPOCV, leave-one-participant-out cross-validation. (TIF) [file pbio.3002732.s003.tif]

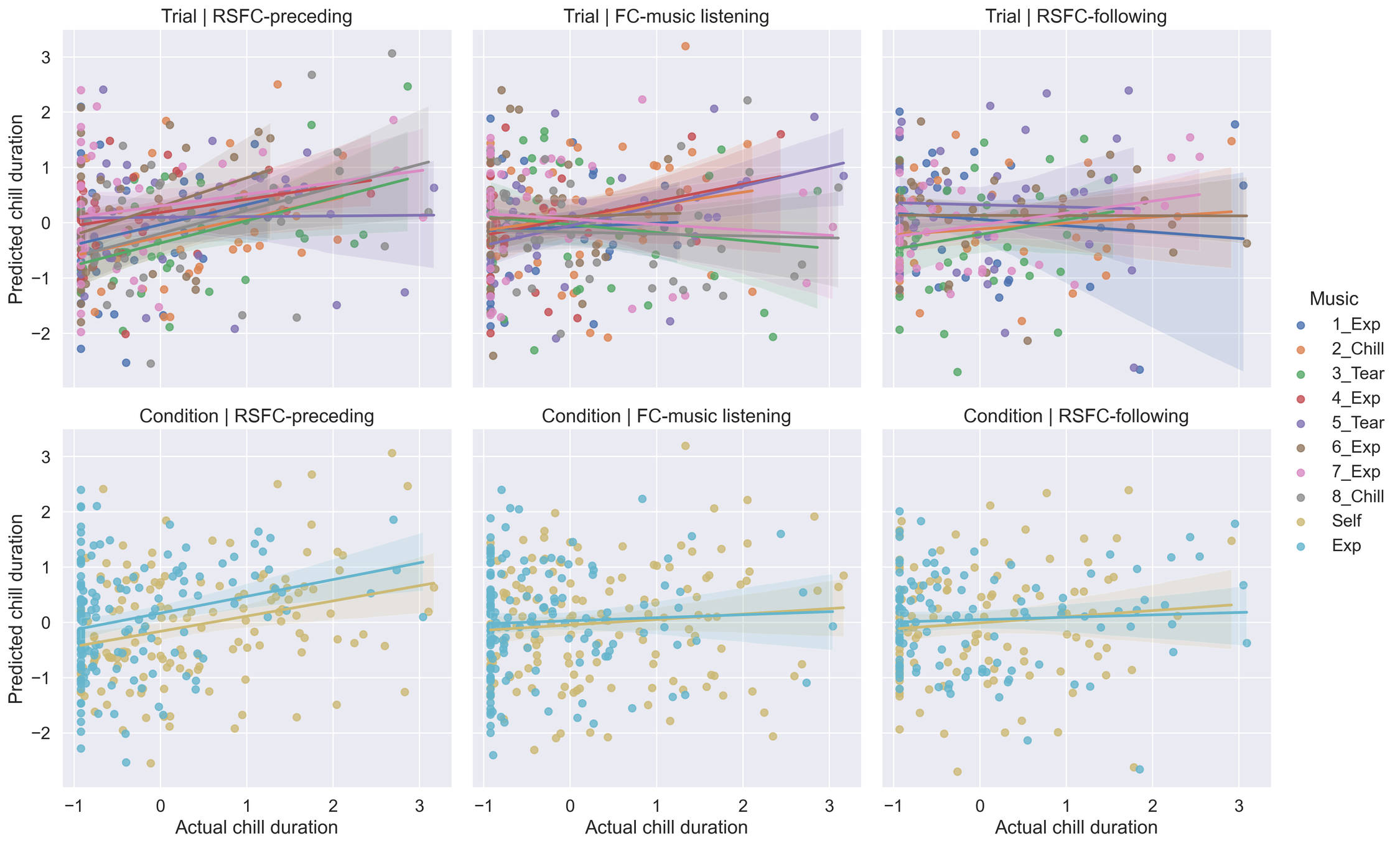

Supplement: S4 Fig — (A) The correlation coefficients were calculated by each music trial. RSFC-preceding, mean r36 = 0.28, SD = 0.13, FC-music listening, mean r36 = 0.09, SD = 0.19, RSFC-following, mean r36 = 0.08, SD = 0.14. Predictive performance varied between trials, but it was difficult to find a specific tendency (e.g., better performance in the first half and worse performance in the second half). Note that for the prediction for each song, machine learning was not performed with data about individual songs. From the machine learning results using data from all trials, we extracted the predicted and measured scores for each song and examined the correlations. (B) The correlation coefficients were calculated by each self- and experimenter-selected music condition. RSFC-preceding, self: r36 = 0.29, experimenter: r36 = 0.25, FC-music listening, self: r36 = 0.10, experimenter: r36 = 0.05, RSFC-following, self: r36 = 0.10, experimenter: r36 = 0.06. Since the correlation coefficient between actual chills and predicted chills was very close for the self- and experimenter-selected music condition, the trial-by-trial machine learning model captured the overall tendency of chill responses regardless of the 2 music types. Each dot represents each trial. Error bands indicate the 95% confidence interval. Exp, experimenter; FC, functional connectivity; LOTOCV, leave-one-trial-out cross-validation; RSFC, resting state functional connectivity. (TIF) [file pbio.3002732.s004.tif]

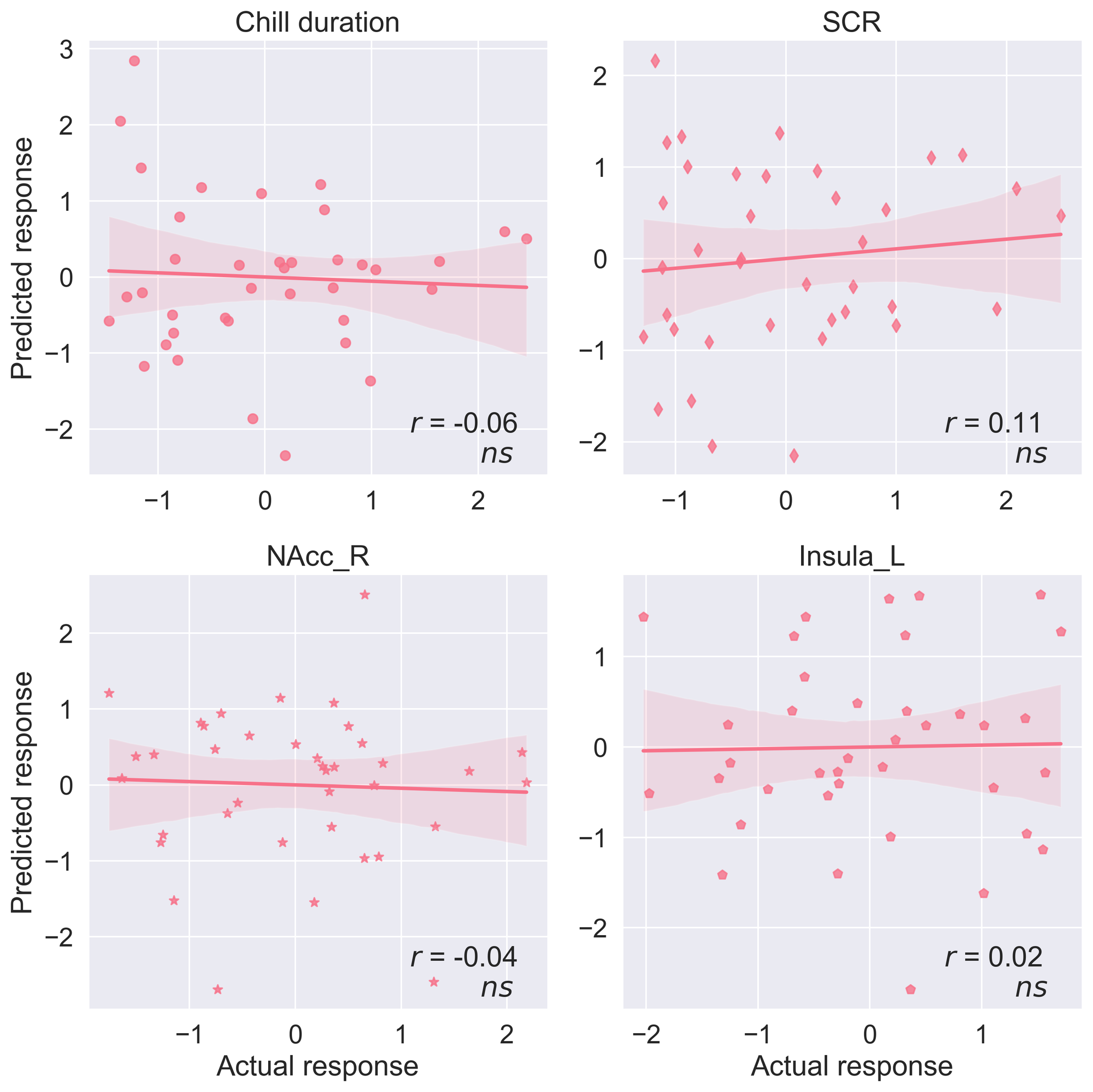

Supplement: S5 Fig — The pre-listening auditory-reward network, as well as right NAcc and left insula activity during the chills experience, was calculated from a time series BOLD signal without the physiological covariates derived from [83] removed. Each dot shows the results of LOPOCV prediction using the auditory-reward brain network. LOPOCV, leave-one-participant-out cross-validation; NAcc, nucleus accumbens. (TIF) [file pbio.3002732.s005.tif]

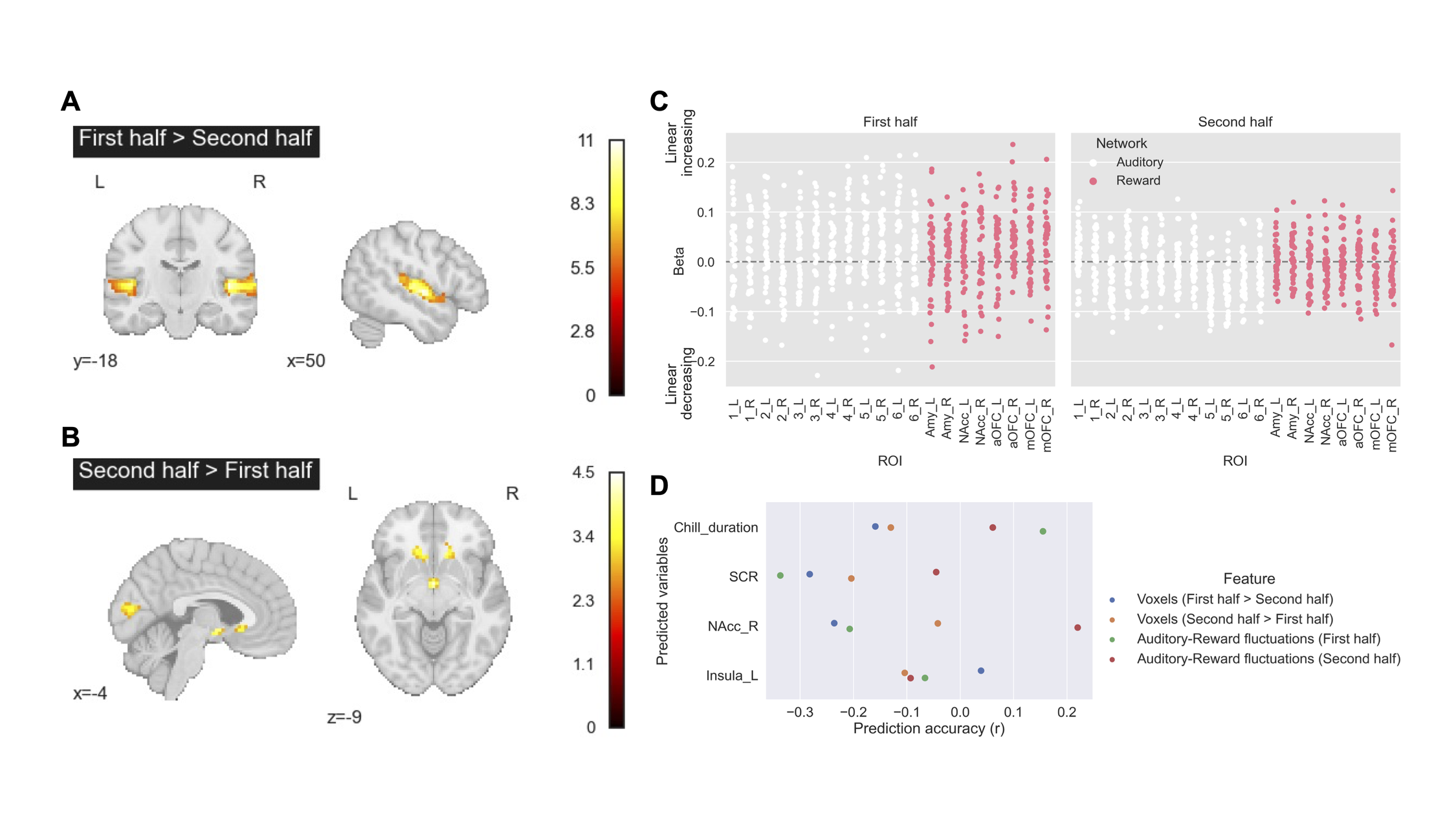

Supplement: S6 Fig — (A) Results of a whole-brain comparison between 2 epochs. The comparison of the first half to the second half revealed greater activity in the bilateral auditory cortical regions (FWE, p < .05). (B) The reverse contrast illustrated greater activity on the vmPFC/NAcc, hypothalamus, and vision regions (uncorrected, p < .005 for display purposes). (C) Time-dependent increasing or decreasing tendencies of BOLD activity (each dot shows each participant). (D) Performance of BOLD activity patterns predictive modeling for chills-related variables using LOPOCV. a, anterior; Amy, amygdala; L, left; LOPOCV, leave-one-participant-out cross-validation; m, medial; NAcc, nucleus accumbens; OFC, orbitofrontal cortex; R, right; SCR, skin conductance response; vmPFC, ventromedial prefrontal cortex. See also S1 Text and S4 Table. (TIF) [file pbio.3002732.s006.tif]

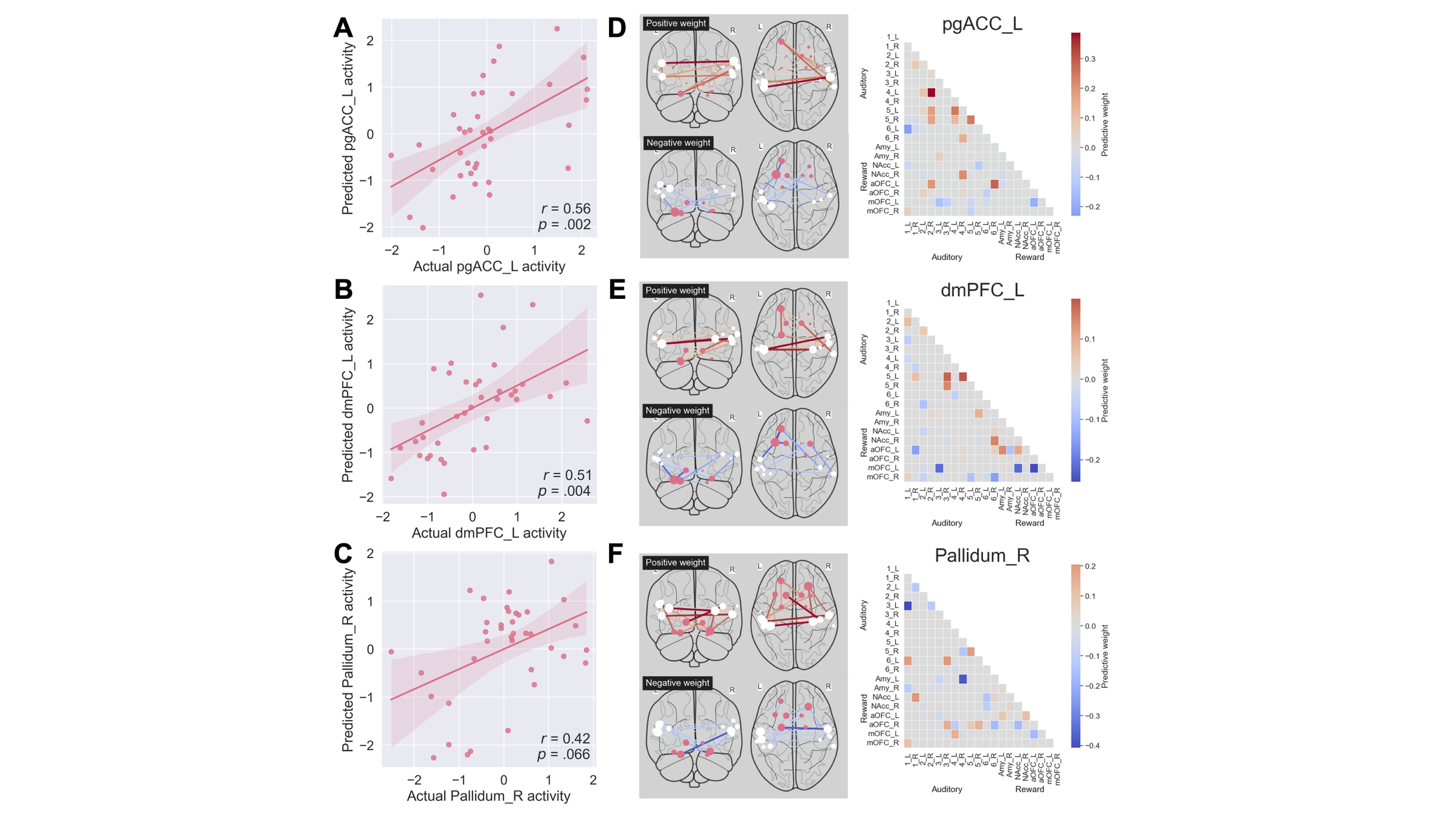

Supplement: S7 Fig — (A, B, C) Prediction accuracy for pgACC, dmPFC, and Pallidum activities during chills experience. The actual versus predicted neural activity by LOPOCV is shown in the plots. (D, E, F) The positive and negative predictive weight of auditory-reward RSFC for pgACC, dmPFC, and Pallidum. dmPFC, dorsomedial prefrontal cortex; L, left; LOPOCV, leave-one-participant-out cross-validation; pgACC, pregenual anterior cingulate cortex; R, right. Note that we show left pgACC, dmPFC, and Pallidum prediction plots in the Supporting information since these predictions did not generalize in Experiment 2 (see also S10 Fig). (TIF) [file pbio.3002732.s007.tif]

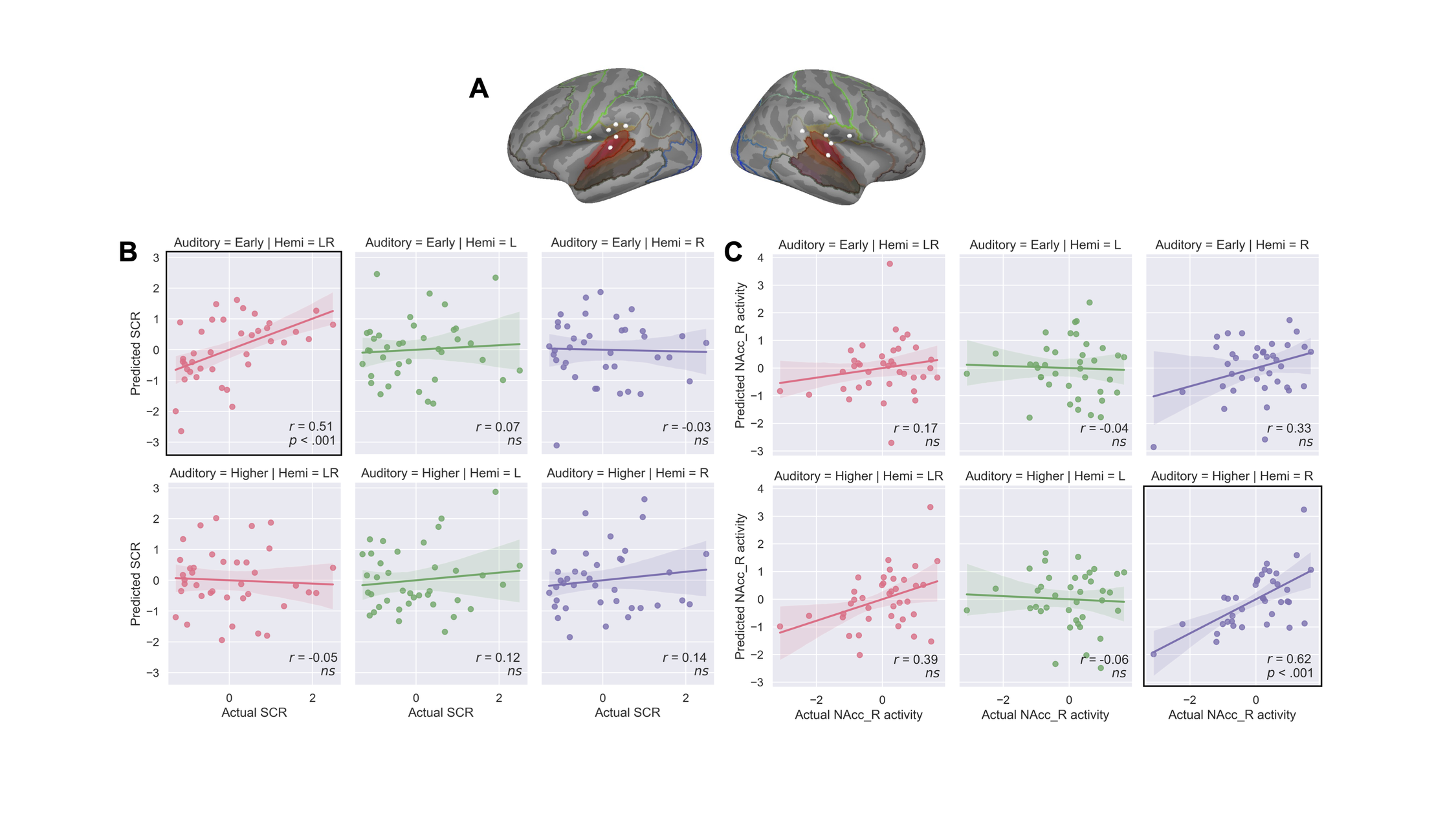

Supplement: S8 Fig — (A) The early (red) and higher-order (brown) auditory regions from HCP [48] and auditory network (white) from [44]. Using the early or higher-order auditory ROI from [48] and reward network from [44], we performed LOPOCV analysis. (B) Prediction accuracy for (B) SCR intensities and (C) NAcc activities during chills experience as a function of bilateral, left, and right brain networks and the 2 types of auditory ROI. HCP, human connectome project; L, left; LR, left and right; LOPOCV, leave-one-participant-out cross-validation; NAcc, nucleus accumbens; R, right; ROI, region of interest; SCR, skin conductance response. (TIF) [file pbio.3002732.s008.tif]

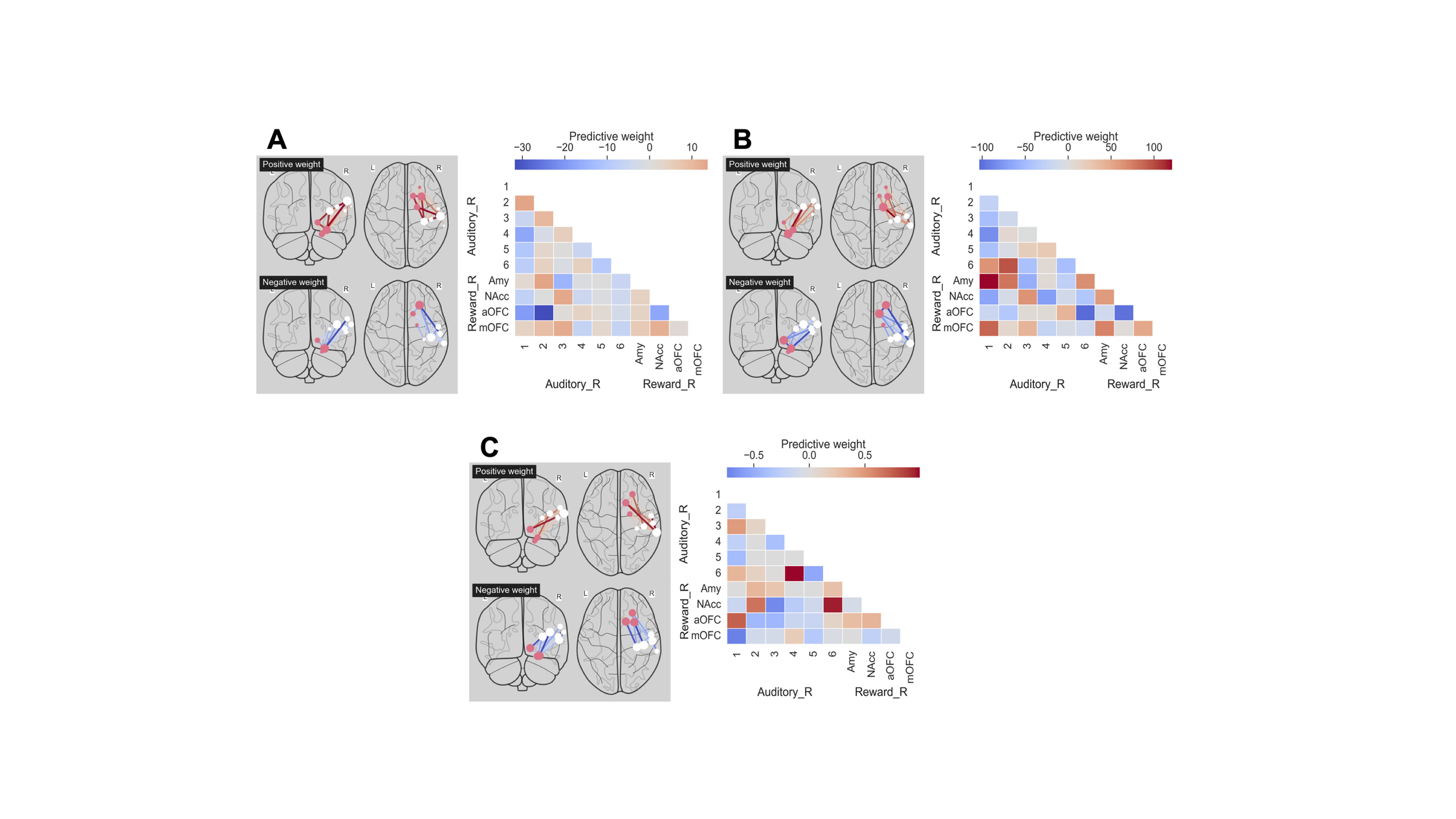

Supplement: S9 Fig — (A) Subjective duration, (B) SCR intensity, and (C) right NAcc activity of chills. Nodes are sized and colored as in Fig 5. Predictive weights showed a similar tendency as the bilateral hemisphere weight. Auditory-amygdala connection showed a higher weight for SCR, whereas the auditory-NAcc/OFC connection showed a higher weight for right NAcc. a, anterior; Amy, amygdala; L, left; NAcc, nucleus accumbens; m, medial; OFC, orbitofrontal cortex; R, right; RSFC, resting state functional connectivity; SCR, skin conductance response. (TIF) [file pbio.3002732.s009.tif]

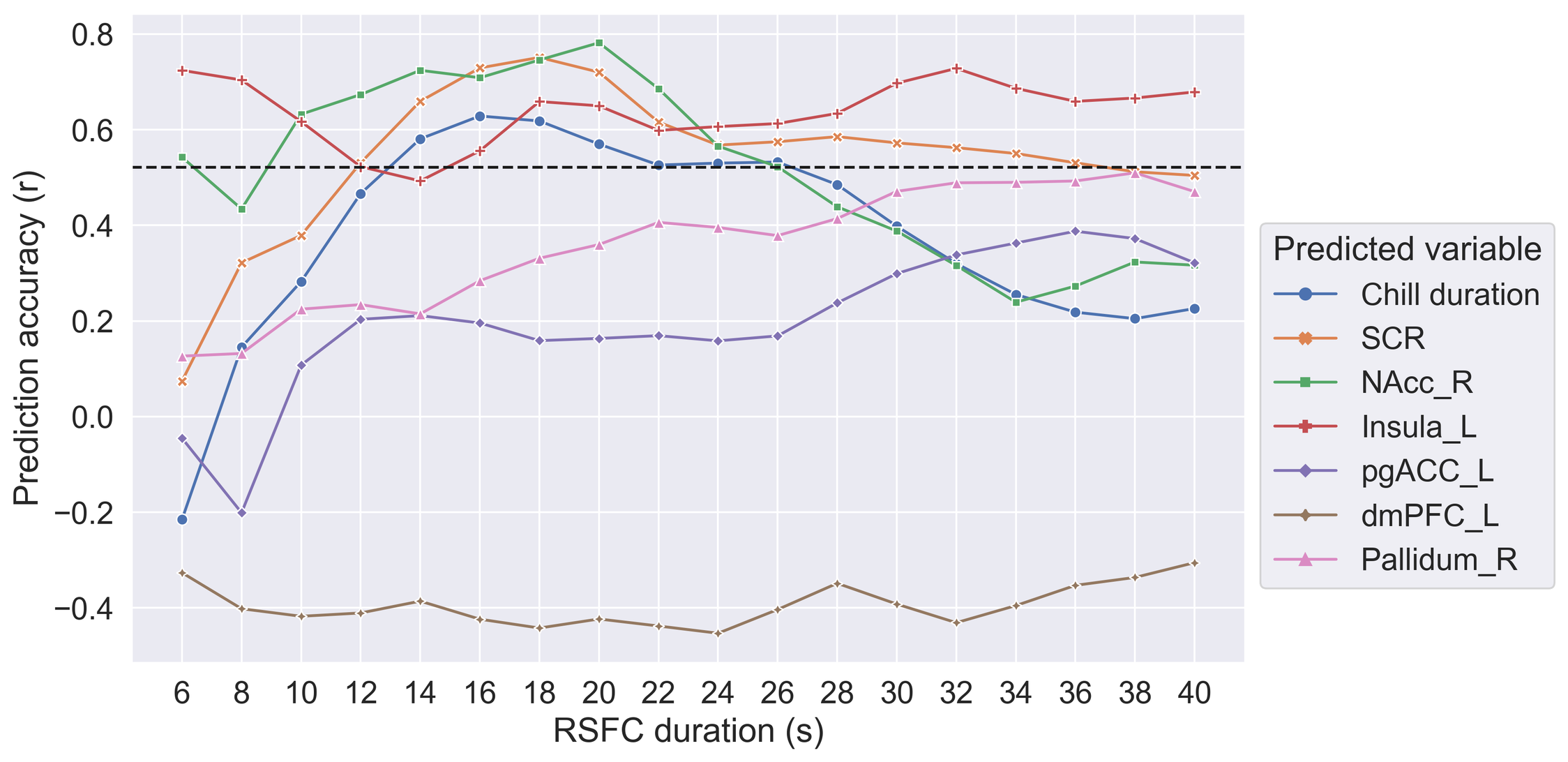

Supplement: S10 Fig — For each chills-related variable, we computed the accuracy of predicted responses from Experiment 1’s machine learning model to predict actual responses obtained by Experiment 2. dmPFC, dorsomedial prefrontal cortex; L, left; NAcc, nucleus accumbens; pgACC, pregenual anterior cingulate cortex; R, right; RSFC, resting state functional connectivity; SCR, skin conductance response. (TIF) [file pbio.3002732.s010.tif]
