# Supplementary material for: State-dependent connectivity in auditory-reward networks predicts peak pleasure experiences to music
Source: PLoS Biol. 2024 Aug 12;22(8):e3002732. doi: 10.1371/journal.pbio.3002732 (PMC11318860; doi:10.1371/journal.pbio.3002732)
Supplement: S1 Table — Fixed 4 experimenter-selected songs for Experiment 1. The selection was based on the 5-download rankings in Japan in 2017. (HTML) [file pbio.3002732.s011.html]

| Artist | Title | Duration | Oricon | TSUTAYA | Recochoku | mora | music.jp |
| --- | --- | --- | --- | --- | --- | --- | --- |
| Gen Hoshino | Love | 253 | 1 | 1 | 1 | 1 | 1 |
| WANIMA | Let's try it | 170 | 2 | 2 | 6 | 3 | 2 |
| DAOKO×Kenshi Yonezu | Fireworks | 270 | 3 | 4 | 7 | 2 | 4 |
| back number | Happy end | 270 | 8 | 7 | 3 | 6 | 3 |
