# Supplementary material for: State-dependent connectivity in auditory-reward networks predicts peak pleasure experiences to music
Source: PLoS Biol. 2024 Aug 12;22(8):e3002732. doi: 10.1371/journal.pbio.3002732 (PMC11318860; doi:10.1371/journal.pbio.3002732)
Supplement: S2 Table — Songs were selected for Experiment 1 in this study. Each participant selected 4 pieces of music. (HTML) [file pbio.3002732.s012.html]

| Participant | Artist | Title | Condition |
| --- | --- | --- | --- |
| 1 | サンボマスター | できっこないを やらなくちゃ | Chill |
| 1 | Baad | 君が好きだと叫びたい | Chill |
| 1 | Bank Band | To U | Tear |
| 1 | Wands | 世界が終るまでは | Tear |
| 2 | フレデリック | オンリーワンダー | Chill |
| 2 | 夜の本気ダンス | Crazy Dancer | Chill |
| 2 | Back Number | クリスマスソング | Tear |
| 2 | Sim | Life Is Beautiful | Tear |
| 3 | よしだたくろう | リンゴ | Chill |
| 3 | 秦 基博 | 鱗 | Chill |
| 3 | スキマスイッチ | 未来花 | Tear |
| 3 | Seamo | Mother | Tear |
| 4 | Mr.children | 終わりなき旅 | Chill |
| 4 | Wanima | Charm | Chill |
| 4 | Kiroro | Best Friend | Tear |
| 4 | ゆず | 栄光の架橋 | Tear |
| 5 | Saucy Dog | いつか | Chill |
| 5 | [Alexandros] | ワタリドリ | Chill |
| 5 | かりゆし58 | アンマー | Tear |
| 5 | Deen | このまま君だけを奪い去りたい | Tear |
| 6 | 江頭勇哉 | ぽろぽろ | Chill |
| 6 | One Ok Rock | Pierce | Chill |
| 6 | 河口恭吾 | 桜 | Tear |
| 6 | Orange Range | Sp Thanx | Tear |
| 7 | 欅坂46 | 不協和音 | Chill |
| 7 | 乃木坂46 | インフルエンサー | Chill |
| 7 | Miwa Featハジ→ | 夜空 | Tear |
| 7 | 三代目 J Soul Brothers From Exile Tribe | C.o.s.m.o.s. ~秋桜~ | Tear |
| 8 | Aimer | 眠りの森 | Chill |
| 8 | Radwimps | 五月の蝿 | Chill |
| 8 | Mrs. Green Apple | 春愁 | Tear |
| 8 | 熊木杏里 | 誕生日 | Tear |
| 9 | Aaa | Next Stage | Chill |
| 9 | Greeeen | 始まりの唄 | Chill |
| 9 | 星野源 | くだらないの中に | Tear |
| 9 | Kana-Boon | 生きてゆく | Tear |
| 10 | Supercell | 君の知らない物語 | Chill |
| 10 | 米津玄師 | Lemon | Chill |
| 10 | Kokia | たった一つの想い | Tear |
| 10 | 山崎まさよし | One More Time, One More Chance | Tear |
| 11 | フレデリック | オンリーワンダー | Chill |
| 11 | Man With A Mission | フォーカスライト | Chill |
| 11 | Greeeen | 遥か | Tear |
| 11 | Radwimps | スパークル | Tear |
| 12 | The Yellow Monkey | プライマル。 | Chill |
| 12 | 戸川純 | 諦念プシガンガ | Chill |
| 12 | 大森靖子 | マジックミラー | Tear |
| 12 | Mili | Yubikiri-Genman | Tear |
| 13 | One Ok Rock | キミシダイ列車 | Chill |
| 13 | Bump Of Chicken | 流星群 | Chill |
| 13 | 米津玄師 | Lemon | Tear |
| 13 | 槇原敬之 | 僕が一番欲しかったもの | Tear |
| 14 | 宇多田ヒカル | Flavor Of Life | Chill |
| 14 | 平原綾香 | Jupiter | Chill |
| 14 | 植村花菜 | トイレの神様 | Tear |
| 14 | Mr.children | Tomorrow Never Knows | Tear |
| 15 | Uverworld | The Over | Chill |
| 15 | 中孝介 | 花 | Chill |
| 15 | Radwimps | スパークル | Tear |
| 15 | Aqua Timez | エデン | Tear |
| 16 | Lisa | Oath Sign | Chill |
| 16 | ロードオブメジャー | 心絵 | Chill |
| 16 | Rythem | ホウキ雲 | Tear |
| 16 | Zard | Get U're Dream | Tear |
| 17 | Superfly | 愛をこめて花束を | Chill |
| 17 | Supercell | 君の知らない物語 | Chill |
| 17 | Bump Of Chiken | ダンデライオン | Tear |
| 17 | Little Glee Monster | ファイト！ | Tear |
| 18 | Sumika | フィクション | Chill |
| 18 | Official髭男Dism | ノーダウト | Chill |
| 18 | 福山雅治 | 零 -Zero- | Tear |
| 18 | Mr.children | Another Story | Tear |
| 19 | 安室奈美恵 | Hero | Chill |
| 19 | 竹原ピストル | よー、そこの若いの | Chill |
| 19 | 椎名林檎 | ありあまる富 | Tear |
| 19 | 手嶌葵 | 明日への手紙 | Tear |
| 20 | レミオロメン | もっと遠くへ | Chill |
| 20 | スキマスイッチ | マリンスノウ | Chill |
| 20 | Aiko | 恋をしたのは | Tear |
| 20 | Yui | To Mother | Tear |
| 21 | モンゴル800 | あなたに | Chill |
| 21 | Flumpool | 証 | Chill |
| 21 | ウルフルズ | 笑えれば | Tear |
| 21 | ガガガSp | 線香花火 | Tear |
| 22 | Exile | Everything | Chill |
| 22 | ［Alexandros］ | ワタリドリ | Chill |
| 22 | Sekai No Owari | サザンカ | Tear |
| 22 | コブクロ | Miss You | Tear |
| 23 | 東京事変 | 絶体絶命 | Chill |
| 23 | 東京事変 | メトロ | Chill |
| 23 | 宇多田ヒカル | Kiss & Cry | Tear |
| 23 | 宇多田ヒカル | 道 | Tear |
| 24 | 中島みゆき | たかが愛 | Chill |
| 24 | Garnet Crow | 夢のひとつ | Chill |
| 24 | Misia | 逢いたくていま | Tear |
| 24 | 米津玄師 | Lemon | Tear |
| 25 | Mr. Children | シーソーゲーム | Chill |
| 25 | Alexandros | ワタリドリ | Chill |
| 25 | Aiko | カブトムシ | Tear |
| 25 | 高橋優 | 福笑い | Tear |
| 26 | フラワーカンパニーズ | 深夜高速 | Chill |
| 26 | 上白石萌音 | なんでもないや | Chill |
| 26 | Miwa | Jexxxa | Tear |
| 26 | サザンオールスターズ | 栄光の男 | Tear |
| 27 | Mr.children | 終わりなき旅 | Chill |
| 27 | ゆず | 栄光の架橋 | Chill |
| 27 | Miwa | オトシモノ | Tear |
| 27 | いきものがかり | Sakura | Tear |
| 28 | Unizon Square Garden | 天国と地獄 | Chill |
| 28 | Orange Renge | イケナイ太陽 | Chill |
| 28 | 東方神起 | Stand By U | Tear |
| 28 | 咲妃みゆ | 灰色の午後 | Tear |
| 29 | 平井堅 | 瞳をとじて | Chill |
| 29 | 一青窈 | ハナミズキ | Chill |
| 29 | スピッツ | チェリー | Tear |
| 29 | Zard | 負けないで | Tear |
| 30 | Burnout Syn Dromes | 君をアンインストールできたなら | Chill |
| 30 | Superfly | タマシイレボリューション | Chill |
| 30 | Kana-Boon | 東京 | Tear |
| 30 | 乃木坂46 | きっかけ | Tear |
| 31 | Monoeyes | 明日公園で | Chill |
| 31 | Uverworld | ゼロの答 | Chill |
| 31 | 中島みゆき | 糸 | Tear |
| 31 | 飯田舞 | キミの隣で… | Tear |
| 32 | B’z | ギリギリChop | Chill |
| 32 | Glim Spanky | 闇に目を凝らせば | Chill |
| 32 | 桑田佳祐 | 白い恋人達 | Tear |
| 32 | スピッツ | ロビンソン | Tear |
| 33 | 加賀美セイラ | Follow Me Feat.sound Around | Chill |
| 33 | Day After Tomorrow | そして僕にできるコト | Chill |
| 33 | スキマスイッチ | 奏 | Tear |
| 33 | Hy | Nao | Tear |
| 34 | Nico Touches The Walls | 夏の大三角形 | Chill |
| 34 | Amazarashi | 空に歌えば | Chill |
| 34 | スキマスイッチ | 藍 | Tear |
| 34 | Bump Of Chicken | K | Tear |
| 35 | Fozztone | Shangri-La | Chill |
| 35 | フジファブリック | 赤黄色の金木犀 | Chill |
| 35 | フラワーカンパニーズ | 深夜高速 | Tear |
| 35 | 10-Feet | 淋しさに火をくべ | Tear |
| 36 | 福山雅治 | 家族になろうよ | Chill |
| 36 | Greeeen | いつまでも | Chill |
| 36 | 平井堅 | 君の好きなとこ | Tear |
| 36 | サザンオールスターズ | Tsunami | Tear |
| 37 | 鬼束ちひろ | 月光 | Chill |
| 37 | 宇多田ヒカル、椎名林檎 | 二時間だけのバカンス | Chill |
| 37 | 宇多田ヒカル | 初恋 | Tear |
| 37 | アンジェラアキ | 手紙 ~拝啓 十五の君へ~ | Tear |
| 38 | Mrs.green Apple | Start | Chill |
| 38 | Superfly | 愛をこめて花束を | Chill |
| 38 | 平井堅 | ノンフィクション | Tear |
| 38 | けやき坂46 | イマニミテイロ | Tear |
