# Supplementary material for: State-dependent connectivity in auditory-reward networks predicts peak pleasure experiences to music
Source: PLoS Biol. 2024 Aug 12;22(8):e3002732. doi: 10.1371/journal.pbio.3002732 (PMC11318860; doi:10.1371/journal.pbio.3002732)
Supplement: S3 Table — Songs were selected for Experiment 2 in this study. Each participant selected 4 pieces of music. (HTML) [file pbio.3002732.s013.html]

| Participant | Artist | Title | Condition |
| --- | --- | --- | --- |
| 1 | 夜の本気ダンス | Take My Hand | Chill |
| 1 | Mrs. Green Apple | Speaking | Chill |
| 1 | 米津玄師 | アイネクライネ | Tear |
| 1 | サカナクション | 目が明く藍色 | Tear |
| 2 | Exile | 道 | Chill |
| 2 | Greeeen | キセキ | Chill |
| 2 | Exile | ただ...逢いたくて | Tear |
| 2 | Boa | メリクリ | Tear |
| 3 | Uverworld | 一億分の一の小説 | Chill |
| 3 | Scandal | 瞬間センチメンタル | Chill |
| 3 | ツリメラ | 氷結 | Tear |
| 3 | 天野月子 | 聲 | Tear |
| 4 | Back Number | ヒロイン | Chill |
| 4 | Uverworld | ナノセカンド | Chill |
| 4 | Mr.children | 花　-Memento-Mori- | Tear |
| 4 | スピッツ | 魔女旅に出る | Tear |
| 5 | 欅坂46 | 不協和音 | Chill |
| 5 | やくしまるえつこ | ニュームーンに恋して | Chill |
| 5 | 嵐 | Pika☆☆Nchi Double | Tear |
| 5 | Shishamo | 君とゲレンデ | Tear |
| 6 | Jam Project | The Hero!! 〜怒れる拳に火をつけろ〜 | Chill |
| 6 | Linked Horizon | 心臓を捧げよ！ | Chill |
| 6 | 石鹸屋 | ドッグハート | Tear |
| 6 | 岸田教団& The 明星ロケッツ | Live My Life | Tear |
| 7 | B'z | Ultra Soul | Chill |
| 7 | 本間芽衣子、安城鳴子、鶴見知利子 | Secret Base〜君がくれたもの〜 (10 Years After Ver.) | Chill |
| 7 | Perfume | Spring Of Life | Tear |
| 7 | Funky Monkey Babys | あとひとつ | Tear |
| 8 | 東京事変 | 群青日和 | Chill |
| 8 | Wanima | ともに | Chill |
| 8 | コブクロ | Million Films | Tear |
| 8 | Hy | Am11:00 | Tear |
| 9 | カラーボトル | 情熱のうた | Chill |
| 9 | 氣志團 | スウィンギン・ニッポン | Chill |
| 9 | いきものがかり | 茜色の約束 | Tear |
| 9 | 乃木坂46 | きっかけ | Tear |
| 10 | Funky Monkey Babys | 告白 | Chill |
| 10 | Mongol800 | 小さな恋のうた | Chill |
| 10 | Back Number | ささえる人の歌 | Tear |
| 10 | Radwimps | もしも | Tear |
| 11 | May'nと中島愛 | ライオン | Chill |
| 11 | Superfly | 愛をこめて花束を | Chill |
| 11 | 倉木麻衣 | Time After Time ～花舞う街で～ | Tear |
| 11 | Ai | Story | Tear |
