# Supplementary material for: State-dependent connectivity in auditory-reward networks predicts peak pleasure experiences to music
Source: PLoS Biol. 2024 Aug 12;22(8):e3002732. doi: 10.1371/journal.pbio.3002732 (PMC11318860; doi:10.1371/journal.pbio.3002732)
Supplement: S4 Table — Regions of a whole-brain univariate GLM analysis showing significantly different BOLD responses between the first and second half epochs. aAll regions are statistically significant at p < .05 after whole-brain correction for family-wise error multiple comparisons. bVoxels at a voxel-wise significance threshold of uncorrected p < .001. cThis table reports all clusters containing ≧4 voxels, and cluster size indicates the number of voxels at an isotropic resolution of 3 mm. The coordinates refer to MNI space. (DOCX) [file pbio.3002732.s014.docx]

| First half > Second half regions | Cluster size^a, c^ | *x* | *y* | *z* | Peak *t*-value |
| --- | --- | --- | --- | --- | --- |
| Auditory cortex | 490 | -45 | -21 | 6 | 11.00 |
|  | 466 | 51 | -9 | 0 | 11.09 |
| Frontal eye field | 4 | -6 | 27 | 45 | 6.03 |

| Second half > First half regions | Cluster size^b,c^ | *x* | *y* | *z* | Peak *t*-value |
| --- | --- | --- | --- | --- | --- |
| Hypothalamus | 10 | 0 | -3 | -9 | 4.52 |
| Visual cortex | 90 | -15 | -90 | 18 | 4.20 |
|  | 12 | 21 | -87 | 27 | 4.06 |
| Ventromedial prefrontal cortex/Nucleus accumbens | 4 | 12 | 24 | -9 | 3.95 |
|  | 5 | -15 | 27 | -9 | 3.80 |
|  | 4 | 6 | 24 | -6 | 3.63 |
