# Supplementary material for: State-dependent connectivity in auditory-reward networks predicts peak pleasure experiences to music
Source: PLoS Biol. 2024 Aug 12;22(8):e3002732. doi: 10.1371/journal.pbio.3002732 (PMC11318860; doi:10.1371/journal.pbio.3002732)
Supplement: S5 Table — (DOCX) [file pbio.3002732.s015.docx]

| Music condition | Neutral | Pleasure | Chill | Tear |
| --- | --- | --- | --- | --- |
| Chill | 19.9 s (36.3) | 133.2 s (60.2) | 76.0 s (56.9) | 30.3 s (44.0) |
| Tear | 28.1 s (43.7) | 110.3 s (57.1) | 65.5 s (61.7) | 80.4 s (58.0) |
| Experimenter | 59.0 s (57.1) | 132.6 s (62.8) | 30.7 s (43.6) | 19.7 s (44.5) |
