# Supplementary material for: State-dependent connectivity in auditory-reward networks predicts peak pleasure experiences to music
Source: PLoS Biol. 2024 Aug 12;22(8):e3002732. doi: 10.1371/journal.pbio.3002732 (PMC11318860; doi:10.1371/journal.pbio.3002732)
Supplement: S1 Text — (DOCX) [file pbio.3002732.s016.docx]

**BOLD activity patterns failed to predict chills-related musical reward**

**Results**

Although we showed that the auditory-reward RSFC has predictive ability, our analyses did not determine if this solely depends on connectivity; for example, the resting BOLD activity patterns may also be predictive. To address this question, we examined the two possible aspects of the BOLD activity using the Experiment 1 dataset.

First, we wanted to test if the resting BOLD activity patterns could characterize the mental processes. Although we assumed that participants anticipate the upcoming music during the pre-listening period, the participants may have different mental processes that could be reflected in the BOLD activity and reward predictability. We therefore performed a whole-brain univariate ﻿general linear model (GLM) analysis to characterize the BOLD activation pattern during the resting state before music listening. Because the previous vmPFC ROI analysis (see Figure S1) suggested that the first and second half of the 40 s resting state might show different BOLD activation patterns, we examined brain activation patterns in the resting state in two separate epochs. As shown in Figure S6-A, we found that auditory cortical regions were clearly activated in the first half more than in the second half (FWE, *p* < .05). In contrast, vmPFC/NAcc, hypothalamus, and vision regions were activated in the second half more than in the first half (uncorrected, *p* < .001) (Figure S6-B, detailed regions are in Table S4). Most importantly, these BOLD activation patterns failed to predict chills-related musical reward via the LASSO machine learning analysis (Figure S6-D).

Second, a previous study reported that a few seconds of high pre-stimulus brain activity leads to decreased phasic brain responses to money in the rewarding brain regions (1). It is possible that pre-activation of the BOLD signal inhibits and makes the neural regions refractory to musical reward. Therefore, we examined the musical reward predictability of the pre-activation of auditory/reward brain regions. We performed linear regression analysis to show time-dependent increasing or decreasing levels of BOLD activity separating the first and second half (see above). As shown in Figure S6-C, the fluctuation pattern in pre-listening rest was highly individualized, and some of the participants increased brain activity, whereas others decreased brain activity. Next, we performed machine learning analysis using the increase or decrease tendencies (regression beta) of auditory and reward brain activity as features. Again, a LASSO machine learning model failed to predict chills-related musical reward responses (Figure S6-D).

These results thus underline the importance of auditory-reward connectivity as the most important predictor of subsequent pleasure. A past study also indicated the importance of auditory-reward connectivity beyond BOLD activity for musical reward (2); further, several studies showed that ﻿connectivity-based prediction outperforms activity-based prediction (3, 4). The state-dependent interplay between auditory and reward regions is thus a valuable marker of subsequent musical reward.

**Method**

﻿Using task rest fMRI data in experiment 1, we performed whole-brain univariate GLM analyses by SPM12. ﻿Each participant’s data was modeled with a GLM using a block design. Regressors were convolved with the canonical hemodynamic response function, and the cut-off period for high-pass filtering was 128 s. The nuisance parameters, same as RSFC analyses, were included as covariates of noninterest. First-level summary statistic images were entered in a second-level analysis in which subjects were treated as random effects. We modeled the first and second half resting periods as boxcar functions, each for 20 s. Therefore, we applied a paired samples t-test between them to produce group statistical parametric maps.

To characterize the pre-activate direction of BOLD signals in auditory and reward network brain regions, we performed a linear regression analysis. The analysis was aimed to show time-dependent increasing or decreasing levels of BOLD activity separating the first and second halves. Regression analysis was performed for each auditory and reward region and each eight-task rest period to calculate the regression coefficients, and then the regression coefficients were averaged for each region in each participant.

We performed LASSO machine learning analysis using four types of features: the first-level beta scores of voxels passing statistical criteria for (1) the first and (2) the second half period, and increasing or decreasing tendencies of BOLD activity in auditory and reward network brain regions for (3) the first and (4) the second half period. The detailed machine-learning procedure was the same as the RSFC analysis (see main text).

**Reference**

1. B. Chew, *et al.*, Endogenous fluctuations in the dopaminergic midbrain drive behavioral choice variability. *Proc. Natl. Acad. Sci. U. S. A.* **116**, 18732–18737 (2019).

2. V. N. Salimpoor, *et al.*, Interactions between the nucleus accumbens and auditory cortices predict music reward value. *Science* **340**, 216–219 (2013).

3. J. Gonzalez-Castillo, *et al.*, Tracking ongoing cognition in individuals using brief, whole-brain functional connectivity patterns. *Proc. Natl. Acad. Sci. U. S. A.* **112**, 8762–8767 (2015).

4. S. Sadaghiani, J. B. Poline, A. Kleinschmidtc, M. D’Esposito, Ongoing dynamics in large-scale functional connectivity predict perception. *Proc. Natl. Acad. Sci. U. S. A.* **112**, 8463–8468 (2015).
